# Femtosecond Soft X‑ray Absorption Spectroscopy Identifies Metal-Centered S_1_ Excited State of Cyanocobalamin

**DOI:** 10.1021/jacs.6c01860

**Published:** 2026-04-28

**Authors:** Nahid Ghodrati, Luigi Adriano, Samuel M. Berry, Cammille Carinan, Robert Carley, Yi-Ping Chang, Cyril Danilevski, Christian David, Robin Engel, Natalia Gerasimova, David Hammer, Manuel Harder, Ryan M. Lamb, David Lomidze, Talgat Mamyrbayev, Taylor P. McClain, Alivia Mukherjee, Matteo Porro, Martin Teichmann, Monica Turcato, Joana Valerio, Ru-Pan Wang, Zhong Yin, Andreas Scherz, Nils Huse, James E. Penner-Hahn, Roseanne J. Sension, Loïc Le Guyader, Benjamin E. Van Kuiken

**Affiliations:** † 339694 European XFEL, Holzkoppel 4, 22869 Schenefeld, Germany; ‡ Department of Chemistry, 1259 University of Michigan, 930 N University Avenue, Ann Arbor, Michigan 48109-1055, United States; § 28498 Paul Scherrer Institute, 5232 Villigen, Switzerland; ∥ 28332 Deutsches Elektronen-Synchrotron DESY, Notkestr. 85, 22607 Hamburg, Germany; ⊥ Department of Biophysics, 1259 University of Michigan, 930 N University Avenue, Ann Arbor, Michigan 48109-1055, United States; # Department of Molecular Sciences and Nanosystems, Ca Foscari University of Venice, 30172 Venice, Italy; ∇ Institute for Nanostructure and Solid State Physics, 14915 University of Hamburg, Luruper Chaussee 149, 22761 Hamburg, Germany; ° International Center for Synchrotron Radiation Innovation Smart, 13101 Tohoku University, 980-8577 Sendai, Japan; ◆ Center for Free Electron Laser Science, Luruper Chaussee 149, 22761 Hamburg, Germany; ¶ Department of Physics, University of Michigan, 450 Church Street, Ann Arbor, Michigan 48109-1040, United States

## Abstract

Time-resolved X-ray absorption spectroscopy (TRXAS) at the Co L_3_-edge was used to identify metal-centered (MC) character in the S_1_ excited state of cyanocobalamin (CNCbl). Cobalamins have UV/visible spectra that are dominated by intense corrin-based excitations, but these ligand-centered states energetically overlap with charge transfer and MC excited states that may be populated following photoexcitation. Ultrafast optical and hard X-ray spectroscopy have shown that CNCbl forms a structurally distorted S_1_ state, but these probes lack a clear signature of the S_1_ electronic identity, which theory has suggested is a ligand-to-metal charge transfer (LMCT) state. Femtosecond soft X-ray TRXAS offers greater state-selectivity than many optical or hard X-ray probes but has, so far, been limited to highly concentrated (≥100 mM) samples. A new experimental setup at the European X-ray Free Electron Laser (EuXFEL) that enables studies of sub-10 mM samples and provides ∼100 fs time-resolution is used to measure the TRXAS of CNCbl at the Co L_3_-edge. Comparison of the L_3_-edge XAS spectrum measured at 0.8 ps with ligand field multiplet simulations indicates that the S_1_ state is primarily an MC excited state. The sub-20 μOD detection sensitivity achieved in this study demonstrates the possibility of applying this method to a wide range of naturally occurring and synthetic transition metal complexes.

## Graphical abstract

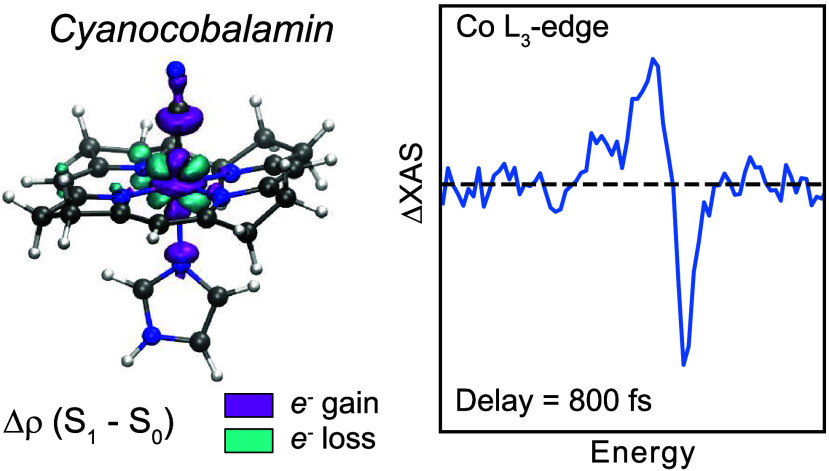

## Introduction

Over the last two decades, time-resolved X-ray absorption spectroscopy (TRXAS) employing hard X-rays (>5000 eV) has become a well-developed technique that permits the study of the ultrafast structural dynamics of transition metal complexes in solution.^1,2^ Experiments may be carried out at synchrotrons and X-ray free electron lasers (XFELs), which typically provide ∼100 ps and ∼100 fs time resolution, respectively. Measurements are now routinely possible on samples containing transition metal atoms with concentrations of only a few mM such as metalloenzymes and synthetic complexes with low solubility or availability.^3–9^ While hard X-ray TRXAS provides transient electronic structure information such as oxidation state changes, it can be difficult to disentangle the electronic and structural components. Soft X-rays (100–1000 eV) offer the possibility of a more direct probe of the electronic structure and higher energy resolution. This range contains the L_2/3_-edges (2p → 3d) providing dipole-allowed transitions to the 3d orbitals for the first row of transition metal elements as well as the K-edges of ligand atoms (C, O, N). Metal L-edge spectroscopy can provide clear signatures of spin, oxidation state, and the chemical bonding environment around the metal.^10–13^ The ligand K-edge can report on metal–ligand covalency.^14^ Despite the potential of these soft X-ray probes, the adoption of soft X-ray TRXAS as a standard tool to investigate the photochemistry of coordination complexes has been hampered by technical hurdles.

The limited use of soft X-ray techniques stems from the strong material absorption in the soft X-ray regime. X-ray penetration depths for liquid samples are on the order of ∼0.5–5 μm depending on the solvent and photon energy, and experiments must be carried out in vacuum environments, which complicates sample handling. In the picosecond time regime, transmission mode XAS with soft X-rays is a well-established synchrotron technique,^15^ which has enabled numerous photophysical and photochemical investigations^16–25^ Synchrotron-based measurements can benefit from a MHz repetition rate source, which provides sufficient count rates to average out fluctuations from liquid jet samples. Moving to femtosecond measurements presents a formidable challenge. One option is the “slicing” technique that has been used to perform femtosecond TRXAS at synchrotrons.^26,27^ However, due to the technical challenges and limited brightness of this method, there is only a single example for a transition metal L-edge measurement, which required a 100 mM sample.^27^ In the case of XFELs, there are a few examples of Fe L-edge and N K-edge XAS, but samples had concentrations ≥300 mM in the absorbing atom to compensate for low count rates due to the 120 Hz repetition rate of the facility.^28,29^ Here, we report a new implementation of femtosecond TRXAS with soft X-rays for dilute liquid samples at a high-repetition rate XFEL. This approach is based on the recently reported beam splitting off-axis zone plate (BOZ)-XAS setup, which has been used to measure solid-state thin-film samples.^30–32^

The BOZ-XAS experimental setup is shown in Figure 1a. Monochromatic X-ray pulses are delivered to the Spectroscopy and Coherent Scattering (SCS) instrument at the SASE3 branch of European XFEL (EuXFEL).^33,34^ These X-ray pulses pass through an elliptical BOZ optical element, which combines a transmission grating and zone plate to both split and focus the beam. The three beams created by the BOZ are detected on a pixel detector, the DSSC,^35^ that reads out the signal from each X-ray pulse. The central beam from the BOZ passes through a flat liquid jet giving the transmitted intensity (*I*_t_), while the side beams freely propagate providing the reference measurements (*I*_0a_ and *I*_0b_). Due to the MHz read-out rate of the DSSC, the setup is able to take full advantage of the high-repetition rate of EuXFEL depicted in Figure 1a. EuXFEL delivers 10 “pulse trains” per second. In the results reported here, each train contained 400 X-ray pulses yielding an effective repetition rate of 4 kHz.

**Figure 1:**
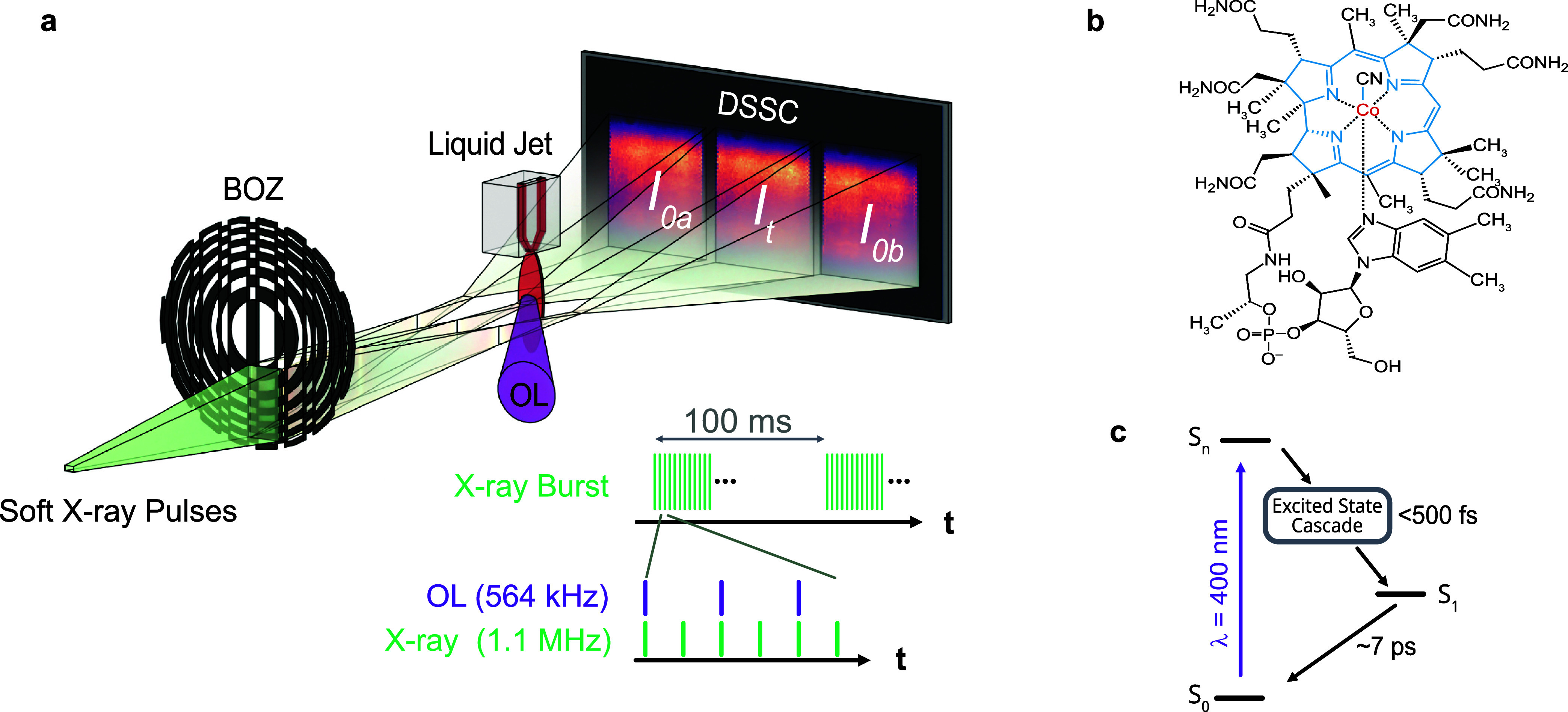
Experimental overview. (a) The BOZ-XAS experimental setup for TRXAS measurements on liquid samples. Monochromatic soft X-rays are delivered by EuXFEL. A beam-splitting off-axis zone plate (BOZ) splits the incident X-ray beam into three copies. The central beam passes through the jet providing a measure of transmitted intensity (*I*_t_). The side beams act as reference measurements (*I*_0a_ and *I*_0b_). The DSSC detector performs shot-to-shot detection. The scheme in the bottom right shows the burst mode pulse pattern of the European XFEL. A 400 nm optical laser (OL) pulse excites the sample for every other X-ray pulse. (b) Molecular structure of CNCbl where the central corrin ring is highlighted in blue. (c) The CNCbl Jablonski diagram.

In the following we demonstrate this new experimental capability by determining the electronic character of the lowest-lying excited state of cyanocobalamin (CNCbl), a representative cobalamin complex. The cobalamin cofactor is best known for its ground state chemistry,^36^ but also acts as photoswitch controlling carotenoid production in bacteria and is actively investigated for potential photochemical applications.^37^ Figure 1b shows the structure of CNCbl where the low-spin Co^3+^ (3d^6^) atom is bound by the corrin ring and axial ligands. The excited state dynamics of CNCbl have been previously studied by ultrafast optical and hard X-ray techniques.^5,6,38^ Its photophysics can be summarized in the simplified scheme shown in Figure 1c. Light absorption creates a corrin-centered π → π* excited state (S_*n*_), which undergoes internal conversion to a structurally distorted state, S_1_, within a few hundred fs. The S_1_ geometric structure exhibits ∼0.2 Å elongation of the axial Co–C and Co–N bonds, which indicates the population of the Co d_*z* ^2^_ orbital. Time-dependent density functional theory (TDDFT) calculations have suggested that this state is a corrin π → Co d_*z* ^2^_ ligand to metal charge transfer (LMCT) state.^39^ However, the Co d_*z* ^2^_ orbital could also be populated via a metal-centered (MC) excitation (3d_π_ → d_*z* ^2^_). The L_3_-edge TRXAS provides a sensitive observable to characterize the Co 3d orbital occupations and consequently the S_1_ state identity. First, we present the TRXAS measurements demonstrating the high-sensitivity of the BOZ-XAS setup. Next, L-edge XAS is compared with ligand field multiplet theory (LMFT) simulations, which indicate that the S_1_ state is predominantly a MC rather than LMCT excited state. This conclusion is further supported by new TDDFT calculations performed at the excited state geometry.

## Results and Discussion

Figure 2a shows the L_3_-edge XAS of 7 mM cyanocobalamin in water. A zoomed-in view of the solvent-subtracted spectrum is shown in the inset. Laser-off (blue) and laser-on (orange) spectra are plotted separately, but are nearly indistinguishable on the scale of the total absorbance. The total X-ray absorbance has a level of 900 mOD and is almost entirely due to the solvent background (dashed gray line). Using tabulated values of the X-ray absorption cross section of water at 780 eV, this corresponds to a liquid jet thickness of ∼2.7 μm.^40^ The background-subtracted cobalamin absorption is 2 orders of magnitude smaller (see inset axis) with a maximum of 7 mOD at 781.6 eV. The spectrum contains a single sharp feature with a high-energy shoulder. This is consistent with other low-spin six-coordinate Co­(III) complexes.^41,42^ It is noted that the XAS spectrum contains an energy dependent background which can be seen as shallow extrema around 774 and 778 eV. This structure does not affect the time-resolved measurements because it appears in laser-on and laser-off spectra, but it complicates background subtraction of static spectra (see Figure S6).

**Figure 2:**
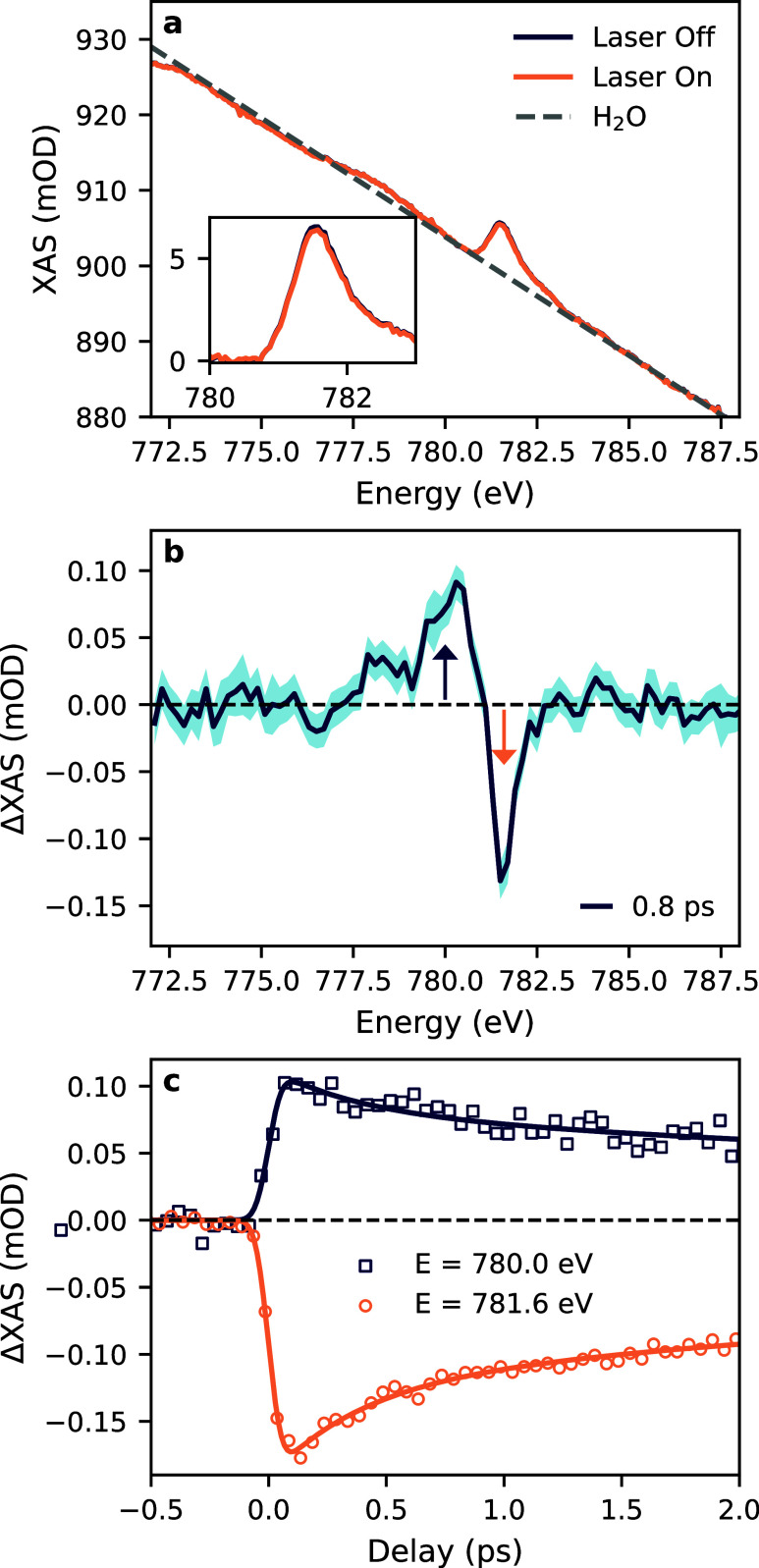
L_3_-edge XAS of Cyanocobalamin. (a) Total absorbance of solvent plus 7 mM cyanocobalamin for laser off (blue) and laser on (orange) measurements. The inset shows a close-up view of the solvent-subtracted L_3_-edge spectrum. (b) TRXAS spectrum measured at a 0.8 ps delay between the 400 nm pump and X-ray probe beam. The shaded region depicts the standard error for each data point, and vertical arrows denote the energies of the delay traces. (c) The time dependence of the transient XAS signal was measured at 780 and 781.6 eV. The solid lines show a simultaneous biexponential fit to the data.

The transient XAS is shown in Figure 2b,c for energy and time scans, respectively. The energy scan collected 0.8 ps after laser excitation exhibits three clear features that rise above the baseline noise. A sharp depletion is observed at the energy of the ground state resonance, and two positive features are centered at 778.2 and 780 eV. The maximum amplitudes of the three features are 0.035, 0.090, and 0.130 mOD. The error bars on the spectrum are given by the standard errors of the measurements, and with the 0.2 eV energy step shown here, the mean error is 12 μOD, which is equivalent to a relative transmission change of <3 × 10^–5^. Delay traces collected at 780.0 and 781.6 eV have been simultaneously fitted with biexponential decays convolved with a Gaussian instrument response function (as described in detail in the SI). The fitted width of the instrument response function yields a time resolution of 106 ± 10 fs fwhm, which validates the ∼100 fs time resolution of the measurement. The long time constant has been fixed to 7.25 ps to match the value determined by optical transient absorption for the ground state recovery for CNCbl at 8 °C.^43^ The 8 °C reference value was used because, although experiments were performed with a room temperature sample reservoir, evaporative cooling from the in-vacuum flat jet is expected to reduce the sample temperature significantly.^44^ The fast time constant was found to be 390 fs. This is attributed to the internal conversion from the Franck–Condon region of the initially ligand-centered excited state through any intermediate states to the S_1_ state. This relaxation time is somewhat longer than the 190 fs time constant previously reported.^5,45^ This difference may be due to measurement factors including excitation wavelength (400 nm vs 520 nm), sample temperature, and choice of excitation energy, but the precision of the fit is also limited by the ∼100 fs time resolution and the sensitivity of the fit to the long time constant (see Figure S7 and Table S1). Overall, the time constant is consistent with the time scales of internal conversion (0.2–0.6 ps) previously reported for cobalamin compounds.^38,43^ The transient spectrum measured at 0.8 ps is temporally separated from the excited state cascade and must be due to the S_1_ electronic state as identified in previous measurements.

We begin by analyzing the character of the S_1_ state by comparing the data with common heuristics for interpreting L-edge XAS. First, the difference in the total spectral intensity between the ground state and excited state is considered by looking at the relative absorption strength of the positive features to the negative features in Figure 2b. The sum of the broad positive bands between 777.5 and 781 eV outweighs the negative contribution from the sharp depletion at 781.6 eV leading to an overall positive integral. This indicates that the excited state possesses a greater spectral intensity than the ground state, which is the opposite of what would be expected for a corrin π → Co d_*z* ^2^_ LMCT state because the integrated L-edge intensity is proportional to the number of vacant 3d orbitals.^46,47^ Second, the excited state spectrum may be constructed from the ground state spectrum and the difference spectrum with knowledge of the excited state fraction as described in the SI. It is found that the shift in the L_3_-edge energy is not significant for any reasonable choice of excited state fraction (see Figure S8c and associated discussion). This also suggests an LMCT S_1_ state is unlikely because typical L-edge shifts associated with oxidation state changes are ∼0.7–1.5 eV.^10,12,41,48^ Instead, this spectrum is consistent with the formation of a d_π_ → d_*z* ^2^_ MC excited state. Such a state has an occupied d_*z* ^2^_ orbital, which is consistent with the axially distorted geometry identified in previous experiments.^5,6^ Further, the formation of a hole in the 3d_π_ and changes in the ligand field strength would give rise to the new low-energy features in the difference spectrum at 778.2 and 780 eV.

LFMT simulations within *D*_4h_ symmetry are used to assess the possibility of an MC excited state. The relative orbital energies of the ligand field model for both the S_0_ and S_1_ states are shown in Figure 3a. The complete set of simulation parameters is given in Table S4. Figure 3b shows the comparison between the simulated and experimental ground state spectrum. The simulation reproduces the single intense absorption feature centered at 781.6 eV. We note that the experimental spectrum is missing intensity in the pre-edge region. This is due to complications with the background subtraction, as discussed in the SI (see S6 and S9). The simulated spectrum of the S_1_ state is shown in Figure 3c together with the experimental S_1_ state constructed for a 5% excitation fraction. There is good agreement between the simulated and experimental spectrum with the most intense feature remaining at 778.6 eV, but new features appearing between 777.5 and 781 eV. As in the ground state, a lack of absorption around 780 eV persists due to the use of the ground state spectrum in constructing the S_1_ spectrum.

**Figure 3:**
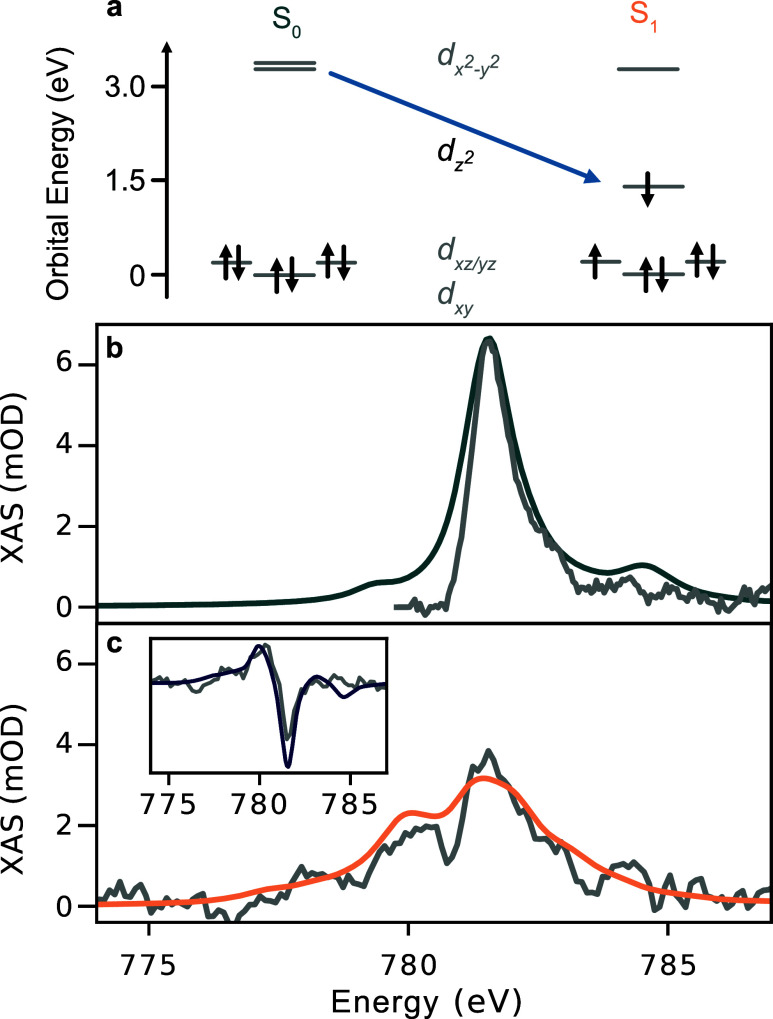
Multiplet simulations of CNCbl XAS. (a) Depicts the energies and occupations of the Co 3d-orbitals in S_0_ and S_1_ states within the approximation of *D*_4h_ symmetry. (b, c) show comparisons of the measured XAS spectrum (gray) with ligand field theoretical simulations (blue) for S_0_ and S_1_ states, respectively. The inset in (c) shows the comparison between the measured and theoretical transient XAS spectrum at 0.8 ps delay.

Although the L-edge XAS is comprised of transitions between the many electron states, the observed XAS lineshapes can be understood in terms of the orbital picture presented in Figure 3a. The ground state possesses a nearly octahedral ligand field with the 3d_σ*_ and 3d_π_ orbitals split by an average of 3.2 eV. Thus, the single intense feature at 781.6 eV is due to 2p → 3d_σ*_ transitions. In the geometrically distorted excited state, the d_*z* ^2^_ orbital energy decreases to 1.4 eV above the energy of the *d*_*xy*_ orbital in the LFMT model as depicted by the arrow in Figure 3a. Thus, the new lower energy features at 780 and 778.2 eV are expected to have significant contributions from excitations to the singly occupied orbitals, d_*z* ^2^_ and either d_*xz*_ or d_*yz*_. The feature at 781.6 eV is unshifted from the ground state, and because there is not a large change in the in-plane ligand field, this peak is attributed to 2p → 3d_*x* ^2^–*y* ^2^_ transitions. These assignments are confirmed by orbital population difference spectra shown in Figure S11, and the chosen ligand field parameters are in good agreement with those predicted by *ab initio* ligand field theory (see SI for details).

The excited states of cobalamin have previously been investigated by TDDFT with the goal of interpreting optical spectra. Kozlowski and co-workers have argued that the BP86 ^49,50^ functional gives the best agreement between theory and experiment by comparison of homogeneously broadened TDDFT transitions and experimental spectra.^51–54^ On the other hand, Brunold and co-workers have found B3LYP^55^ to be suitable.^56,57^ In particular, a recent study suggested that BP86 TDDFT optical spectra suffer from spurious low-energy charge transfer contributions that mimic vibronic structures, and it was concluded that B3LYP provides a better agreement with experiment once the effects of vibrations are included.^57^ Most studies have been carried out at the ground state geometry, except for the work of Lodowski et al., which employed the BP86 functional, explored the excited state potential energy surfaces and found that the S_1_ state was an LMCT state.^39^

Given the ongoing debate over which DFT approximations are most suitable for describing the excited states of cobalamins, we examined the functional dependence of the excited state character at the geometry of the S_1_ state. The calculations were performed on a reduced structural model for CNCbl, [ImCo­(corrin)­CN]^+^, that has commonly been employed in other theoretical studies. Figure 4 shows the character of the S_1_ excited state for various levels of DFT theory at the excited state geometry for BP86, B3LYP, and CAM-B3LYP.^58^ This is represented by the natural transition orbitals (NTOs), which have, in this case, only a single significant (>97%) donor–acceptor orbital pair for the lowest-lying singlet transition. The transitions are further quantified by fragment analysis of electron and hole fractions on the metal and each of the ligands.^59^ Regardless of the functional chosen, the excitation is a mixture of LMCT and MC, with both ligand and metal character for both the donor and acceptor orbitals. The character of the acceptor orbital is independent of functional, which is Co d_*z* ^2^_ with a significant CN^–^lone pair contribution. The hole NTO is a mixture of corrin π and d_*xz*/*yx*_ character. Co exhibits a strong functional dependence that changes the excitation from primarily LMCT for BP86 to MC for the range-separated hybrid CAM-B3LYP. The L_3_-edge XAS is more consistent with the results from the hybrid functionals (B3LYP and CAM-B3LYP), which show a significant MC excited state fraction and are generally expected to provide a more accurate treatment of charge transfer excitations. The hybrid functional results are qualitatively consistent with the multiplet simulations as they exhibit d_*xz*/*yx*_ hole character and a singly occupied d_*z* ^2^_ orbital. Thus, we conclude that the S_1_ state of cobalamin is primarily a MC excited state but still likely possesses some fractional charge transfer from the corrin ring. We note, however, that there is only a moderate change in the charge density on the Co for these cases because charge transfer from the corrin ring is partially delocalized onto the CN^–^ ligand rather than increasing the electron density on the Co atom. This is consistent with the interpretation of the spectrum from the multiplet simulations where no shift in the 2p → d_*x* ^2^–*y* ^2^_ feature is observed. A more precise quantification of the CT would require a more sophisticated theoretical model for computing the XAS spectra. This could take the form of a model Hamiltonian that includes differential π and σ-CT, or alternatively, an *ab initio* approach based on multiconfigurational wave functions. Such investigations are left to future studies.

**Figure 4:**
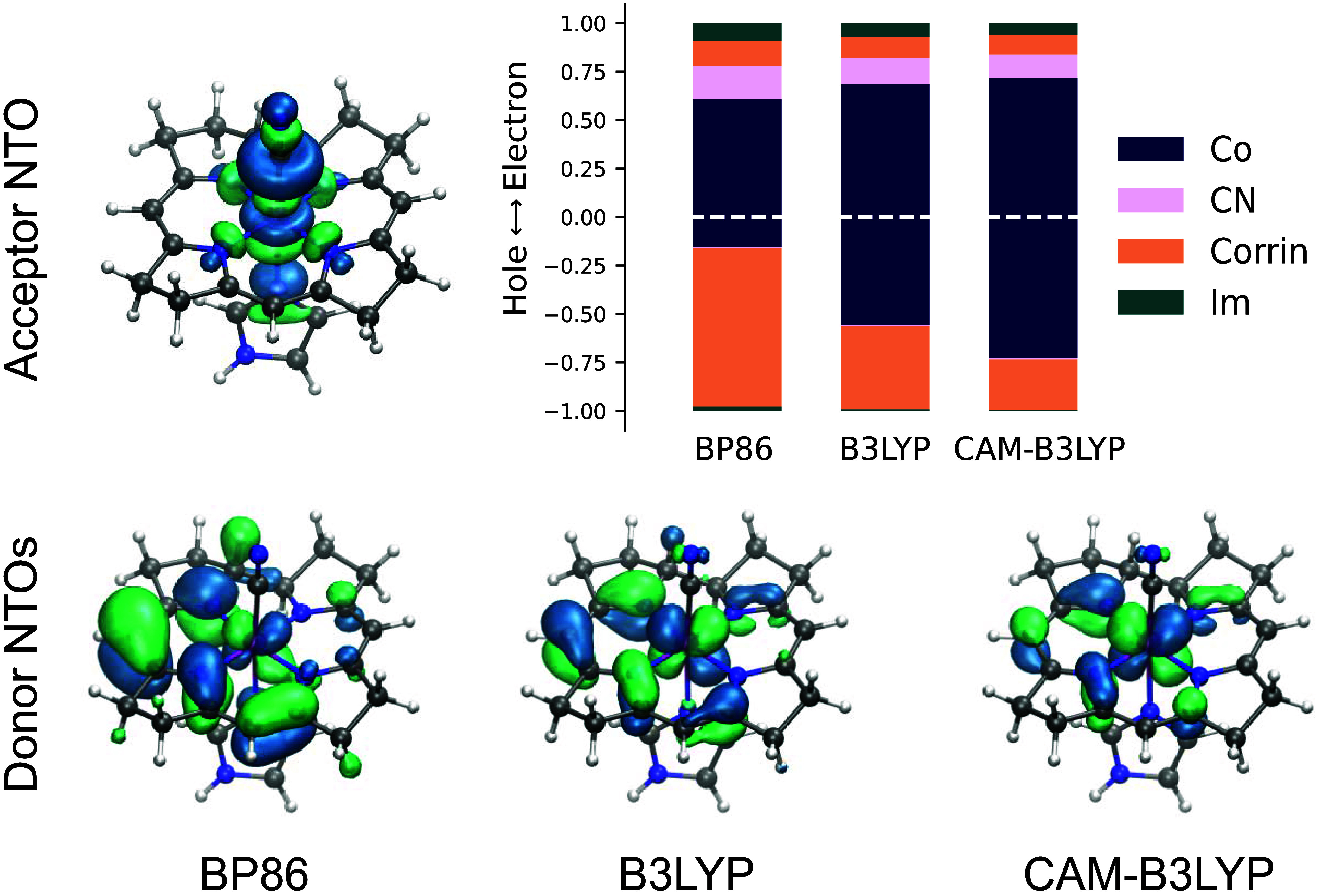
Natural transition orbitals (NTOs) for the S_1_ state computed with BP86, B3LYP, and CAM-B3LYP functionals. The acceptor NTO is similar across all functionals (top left), while the donor NTO (bottom) differs across the series. Fragment analysis of electron and hole fractions is given for the Co center and each ligand at the top right.

The identification of a MC S_1_ excited state for CNCbl has significant implications. First, it suggests that the MC S_1_ excited state exhibiting low-to-moderate CT character leads to rapid ground state recovery and high photostability. Sension et al. also recently identified MC S_1_ excited states in two other common nonalkylcobalamins, hydroxocobalamin (HOCbl) and aquocobalamin (H_2_OCbl^+^).^60^ Excited state deactivation through MC excited states is a common theme in the photophysics of 3d^6^ transition metal complexes,^61^ and it seems that nonaklycobalamins, which are known for their photostability, follow this motif. On the other hand, photoexcitation of alkylcobalamins leads to bond cleavage between Co and an axial ligand and the formation of Co­(II) photoproducts.^37,38^ Identification of electronic states along photochemical pathways in alkylcobalamins such as adenosylcobalamin found in photoreceptor proteins is an active area of research. For example, the transient absorption spectrum of the initial excited state of CarH resembles that of the S_1_ of CNCbl, and this comparison has been used to assign the initial excited state of CarH as LMCT.^62,63^ The L-edge XAS presented here would suggest that the initial excited state of CarH is instead a MC excited state. While this is possible, there are also alternative interpretations of the transient absorption spectroscopy of CarH.^64^ The state selectivity of L-edge TRXAS could potentially be used in future studies to identify the electronic states in these photoactive species.

## Conclusions

Here we have used TRXAS at the L-edge of Co to identify MC character in the CNCbl S_1_ state. These results demonstrate how the state selectivity of L-edge XAS can be used to elucidate photophysical mechanisms in coordination complexes. We emphasize that this study has only been made possible because of the implementation of the BOZ-XAS approach at a high repetition-rate XFEL. The ∼10 mM solubility limit, ultrafast excited state cascade, and the 7 ps ground state recovery time scale necessitated a high signal-to-noise ratio and femtosecond time resolution.

The capability presented in this work greatly changes the scope of chemical problems that can be addressed by soft X-ray spectroscopy for liquid samples. For the time-resolved data presented in Figure 2, each energy and delay scan represents 40 min of measurement or ∼2 h of total experimental time. These data provide an indication of expected data collection times for more dilute samples, and samples with a concentration of 1.75 mM and similar excitation fraction could be investigated with similar S/N within 32 hr of measurement time. This measurement time is accessible with standard beamtime allocations enabling time-resolved soft X-ray studies on many synthetic coordination complexes and some important metallo-enzymes. For example, heme enzymes such as cytochrome c and myoglobin have been studied with ultrafast hard X-ray spectroscopy at concentrations of 3–4 mM.^3,8^ With the continued development of XFELs targeting full MHz operation, the measurement scheme presented here could gain two additional orders of magnitude in data collection speed, paving the way to measuring systems at the 100 μM concentration.

## Supplementary Material



## Notes

Details of the experimental setup and measurement parameters; description of data processing and analysis; theoretical and computational methods with additional analysis (PDF)
