# Supplementary material for: Femtosecond Soft X‑ray Absorption Spectroscopy Identifies Metal-Centered S1 Excited State of Cyanocobalamin
Source: J Am Chem Soc. 2026 Apr 28;148(18):18995–9003. doi: 10.1021/jacs.6c01860 (PMC13185098; doi:10.1021/jacs.6c01860)
Supplement: Supplementary file 1 [file ja6c01860_si_001.pdf]

Supporting Information

Femtosecond Soft X-ray Absorption Spectroscopy Identifies Metal-Centered S_1 Excited State of Cyanocobalamin

Nahid Ghodrati,[†] Luigi Adriano,[†] Samuel M. Berry,[‡] Cammille Carinan,[†] Robert Carley,[†] Yi-Ping Chang,[†] Cyril Danilevski,[†] Christian David,[¶] Robin Engel,[§] Natalia Gerasimova,[†] David Hammer,[†] Manuel Harder,[†] Ryan M. Lamb,[‡] David Lomidze,[†] Talgat Mamyrbayev,[¶] Taylor P. McClain,[‖] Alivia Mukherjee,[‡] Matteo Porro,[†,⊥] Martin Teichmann,[†] Monica Turcato,[†] Joana Valerio,[†] Ru-Pan Wang,[#] Zhong Yin,[@] Andreas Scherz,[†] Nils Huse,[#,△] James E. Penner-Hahn,[‡,‖] Roseanne J. Sension,[‡,∇] Loïc Le Guyader,[*,†] and Benjamin E. Van Kuiken[*,†]

†*European XFEL, Holzkoppel 4, 22869 Schenefeld, Germany*

‡*Department of Chemistry, University of Michigan, 930 N University Avenue, Ann Arbor, Michigan 48109-1055, United States*

¶*Paul Scherrer Institute, 5232 Villigen PSI, Switzerland*

§*Deutsches Elektronen-Synchrotron DESY, Notkestr. 85, 22607, Hamburg, Germany*

‖*Department of Biophysics, University of Michigan, 930 N University Avenue, Ann Arbor, Michigan 48109-1055, United States*

⊥*Department of Molecular Sciences and Nanosystems, Ca Foscari University of Venice, 30172 Venice, Italy*

#*Institute for Nanostructure and Solid State Physics, University of Hamburg, Luruper Chaussee 149, 22761 Hamburg*

@*International Center for Synchrotron Radiation Innovation Smart, Tohoku University, 980-8577 Sendai, Japan*

△*Center for Free Electron Laser Science, Luruper Chaussee 149, 22761 Hamburg, Germany*

∇*Department of Physics, University of Michigan, 450 Church Street, Ann Arbor, Michigan 48109-1040, United States*

E-mail: loic.le.guyader@xfel.eu; benjamin.van.kuiken@xfel.eu

Experimental Methods

Samples Delivery

Cyanocobalamin (CNCbl) was purchased from Sigma-Aldrich (Merck). Samples were prepared by dissolving CNCbl in deionized water to a concentration of 7 mM. The samples were filtered and transported to the beamline. The samples were fed to the vacuum environment using an HPLC pump operating at a flow rate of ∼1.5 ml/min. The liquid jet was generated from a borosilicate glass chip featuring two inlets and three channels reported by Koralek *et al.* that was designed for gas-dynamic sheet generation.[1] In the present work, the central channel of the chip was plugged, and the sample was flowed through the outer channels mimicking a colliding nozzle flat jet design.[2] The sample delivery was operated in a single pass mode, where the sample was collected in a cold trap. The chamber pressure was maintained at $\sim 5 \times 10^{-4}$ mbar during liquid jet operation.

Experimental Setup

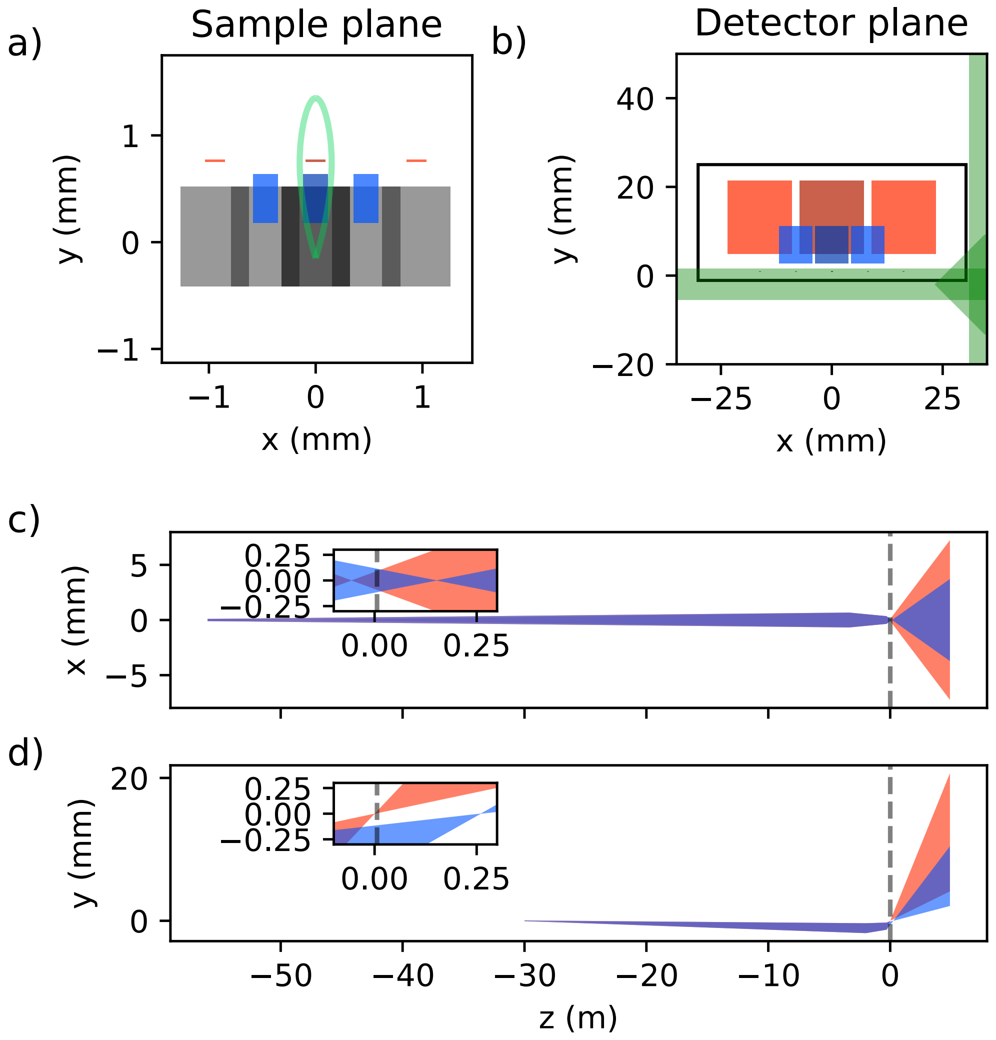

Figure S1: Ray tracing simulation using the *BOZ-calc*[3,4] package of the beams propagation up to (a) the liquid jet and (b) the detector. The focused 1st zone-plate order beam footprint at the respective positions is shown as red for the fundamental 780 eV radiation and blue for the second harmonic 1560 eV. The 0th zone plate order is shown as gray where the darker gray regions between the beams indicate overlaps. The liquid jet is delimited by light green lines. In (b), the DSSC sensor is represented by the black rectangle, and the DSSC filter mount is represented by the green shapes. (c) Horizontal and (d) vertical beam propagation from the source point to the detector. The position of the sample is shown as a dashed vertical line. The inset in each figure shows a zoomed-in region around the sample position. The red-filled areas represent the fundamental 780 eV beams and the blue-filled areas represent the second harmonic 1560 eV beams. The vertical separation between the fundamental and second-harmonic beams after the liquid jet arising from the off-axis component of the diffractive optics can be used to block the second-harmonic from the fundamental.

The setup is similar in many ways to the one described in Ref. 5 which is used for solid targets, and it is shown in Figure 1a of the main text. The key component is a specifically made diffractive optic called an elliptical beam-splitting off-axis zone plate (EBOZ) that splits the incoming FEL beam into three for shot-to-shot normalization while simultaneously focusing the beam onto the jet in the interaction region. The focusing is done with an elliptical zone plate structure which has different focal length horizontally ($f_H = 260\,\mathrm{mm}$) and vertically ($f_V = 320\,\mathrm{mm}$). The vertical focus is close to the liquid jet, while the horizontal focus is upstream of the jet, creating line focus (HxV = $150 \times 10\ \mu m^2$) on the jet. This is to enable refreshing the sample between shots when operating at the MHz rate of EuXFEL. The off-axis component of the zone plate ensures that all the different diffraction orders are vertically separated and non-overlapping on the detector. The beam splitting is realized with a phase grating structure where the phase shift is used to control the intensity ratio between the middle beam (grating 0th order) and the left and right beams ($+1^{\mathrm{st}}$ and -1st grating orders).[6] This allows the intensity of the middle beam to be higher than the left and right reference beams, such that after transmission through the jet, the resulting intensity of each beams is similar. As they propagate after the liquid jet, they expand and cover large non-overlapping areas on the DSSC detector.[7] Illuminating many pixels on the detectors increases the dynamic range of each beam intensity measurement. By maintaining similar intensities, we prevent one beam from saturating the detector while the others produce low signal levels.

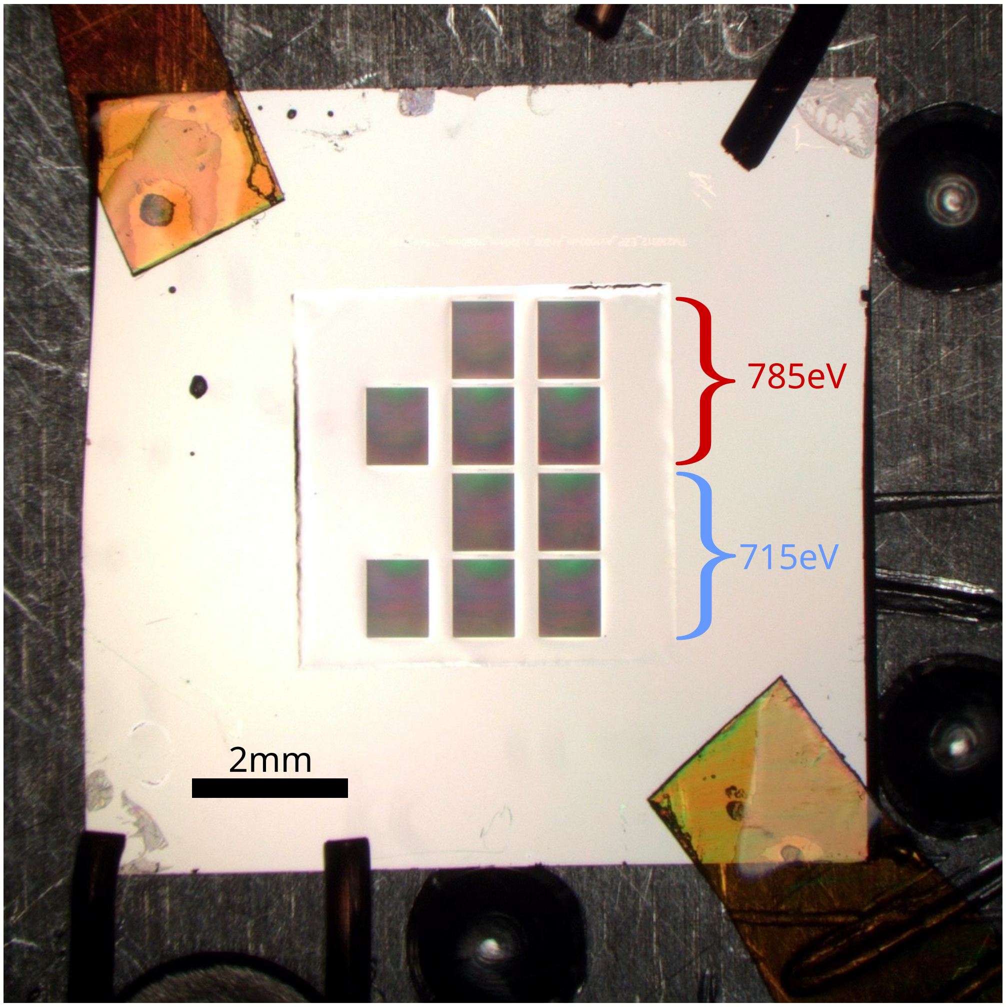

Figure S2: Microscope image of an EBOZ chip. The 10 mm by 10 mm Si chip is etched to form a 5 mm by 5 mm Si membrane with 1 µm thickness. In this membrane, 5 EBOZ for Co at 785 eV and 5 EBOZ for Fe at 715 eV are etched. Each EBOZ has dimensions of HxV = 0.8x1 mm^2. The vertical spacing between EBOZ is 0.1 mm, and the horizontal spacing is 0.3 mm. For each element, the 5 EBOZ have different pattern shifts[6] starting from s = 0.25 in the top left to s = 0.17 in the bottom right, which changes the intensity ratio between 0th and 1st order from 2.5 to 7.0.

Each HxV =0.8x1 mm^2 EBOZ is etched in a 1 µm thick Si membrane which can hold several of them, as shown in Fig. S2. This allows fabrication of a single chip that can provide various intensity ratios between 0th and 1st order beams accommodating various combinations of solvent and jet thickness. One chip can typically hold up to 12 different EBOZ and two chips can be mounted on the in-vacuum manipulator. In-vacuum piezo stages moves 4 independent blades to aperture the X-ray beam in front of the EBOZ to the

appropriate size. The EBOZ chip is mounted on a 4 axis in-vacuum piezo manipulator from SmarAct, with three translation axis, X, Y and Z where Y is vertical and Z is the X-ray beam propagation direction. The fourth axis is a goniometer providing rotation around Z to align the diffracted beam with the DSSC sensor. Motion along Z is required during energy scan to keep the focus at the same place and is performed by a Karabo middle-layer device.

Time-Resolved XAS Measurements

All measurements were performed at the SCS instrument on the SASE3 branch of the European XFEL using the SCS Chem chamber.[8] Monochromatic X-rays were generated by the SASE3 monochromator operating in 2nd order. An EBOZ with a shift value of 0.19 was used, which was designed to give $I_0/I_1 = 5.3$. The X-ray spot size on the sample was measured to be 70 μm in the horizontal direction and 30 μm in the vertical direction. This differs from the designed focused described above. The deviation in the horizontal focus size was due to the X-ray beam being clipped by apertures in the differential pumping section that separates the Chem chamber from the high vacuum sections of the beamline. The mismatch in vertical focus size is attributed to a deviation in the sample position from the design value. XAS spectra were collected by continuously scanning the monochromator energy together with the undulator gaps back and forth over the Co L_3-edge energy range of 772 to 790 eV. The absolute energy axis is set by the beamline calibration to the Ne absorption spectrum.

Optical laser excitation of samples was performed using the XFEL PP laser system. The 800 nm output of the laser system was frequency doubled, and samples were excited using a $\sim$1 μJ pulse focused to a $1/e^2$ width of 41 μm horizontally by 50 μm vertically. Delay scans were made by scanning a mechanical delay stage.

Data Reduction and Analysis

Event filtering

During data collection runs, we noticed that water or ice droplets may fly across one of the beams ruining the normalization quality. These events were determined to be rare enough that event filtering can be effectively used to remove them. One such event is shown in Fig. S3a where the pulse resolved transmission is plotted against the pulse number in the train. The pulse train shown in blue contains the "bad" event, but the next train shown in orange is "normal." We can see that the transmission value is quite similar for the two instances at the start and at the end of the trains. However, in the middle of the first train, there is a large deviation due to the "bad" event. If we select certain DSSC images acquired during the deviation, as shown in the three insets at the start, middle, and end of the deviation in Fig. S3a, we can see a black spot, the shadow cast by an ice particle or droplet, moving across the right beam. To automatically find and filter out these events, we can compute the mean transmission μ and the standard deviation σ for each train, and then build a histogram of σ/μ ratios, as shown in Fig. S3b. We can identify a threshold value (vertical dashed green line) that separates these outlier events from the rest of the data. To confirm the success of this filtering, we then compute the pumped (continuous line) and unpumped (dashed line) XAS on the filtered (orange line) and unfiltered (blue line) data, as shown in Fig. S3c. The result of the filtering is that it removes all the impulsive noise present in the binned XAS data which arises from the outliers created by the ice particles or droplets which dramatically change the X-ray transmission measurement of the jet. The amount of trains filtered out is usually very low, 0.32% of the initial train in this example, which is therefore a negligible fraction.

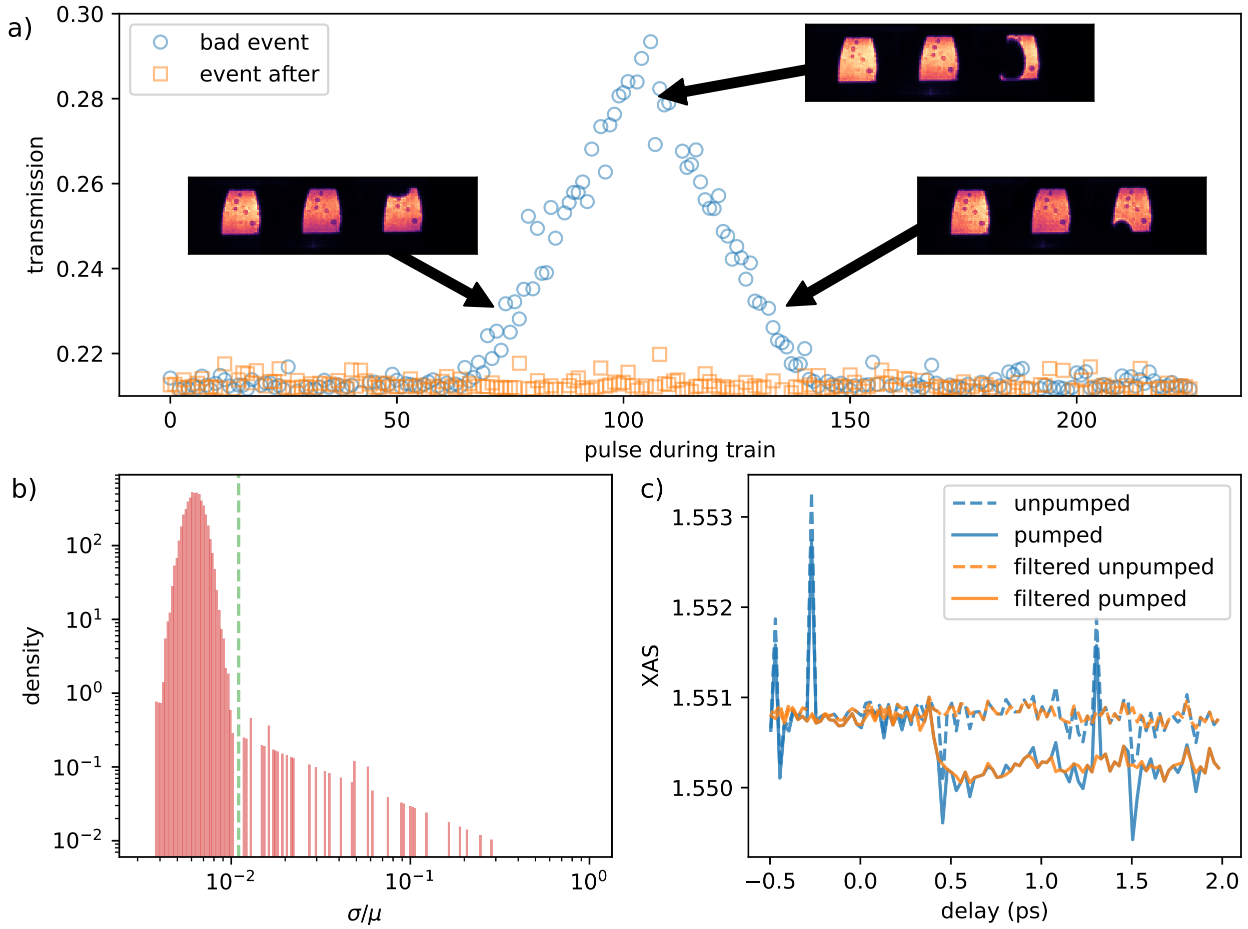

Figure S3: (a) Pulse resolved transmission for a “bad” train and the next train after. The three insets show the collected DSSC image for the “bad” train for the selected pulse in the train, where we can see the particle flying through the right reference beam. (b) Histogram of the train-resolved transmission standard deviation over the mean. (c) XAS of the unpumped in dashed and pumped in continuous line, for the unfiltered in blue and filtered data sets in orange. The complete removal of impulsive noise in the filtered data is visible.

Shot-noise limit

As shown in Ref. 5, the measurement method can, in principle, reach the photon shot-noise limit. In Fig. S4 we plot the single-shot SNR and compare it to the theoretical limit.

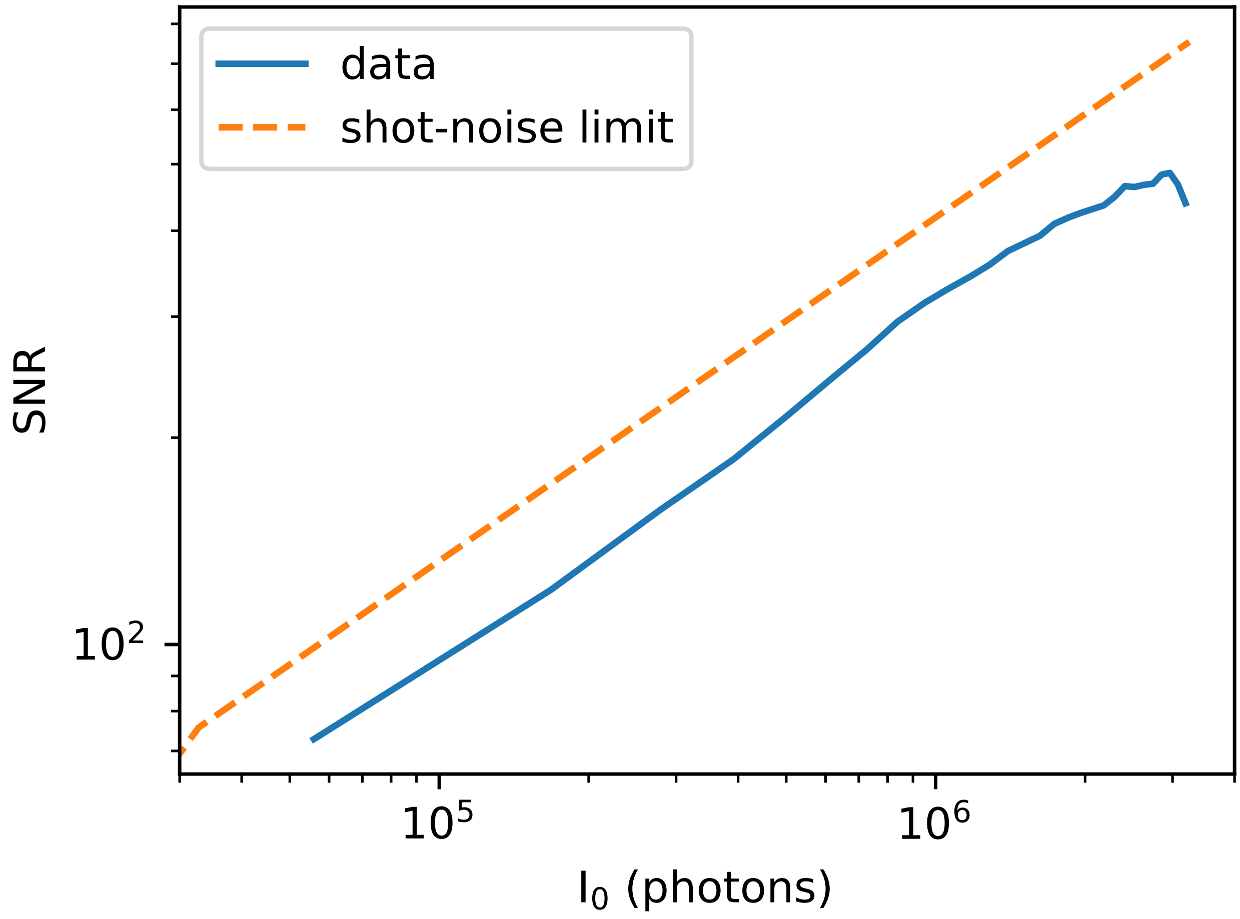

Figure S4: SNR and shot-noise limit as a function of the I_0 intensity.

Jet fluctuation

Fluctuations in the jet thickness over time are not normalized in this measurement scheme. If we look at the measured transmission T_i as a function of the train number i, we ideally expect data to fluctuate around the mean value within the measured uncertainty. In Fig. S5a) we plot the I_0-weighted mean transmission T_i in orange and the I_0-weighted standard error σ_i in blue for 150 consecutive trains. It seems that the data fluctuates more than σ_i. In particular, there is a recognizable pattern where the next transmission T_{i+1} is further away from T_i than T_{i+2}.

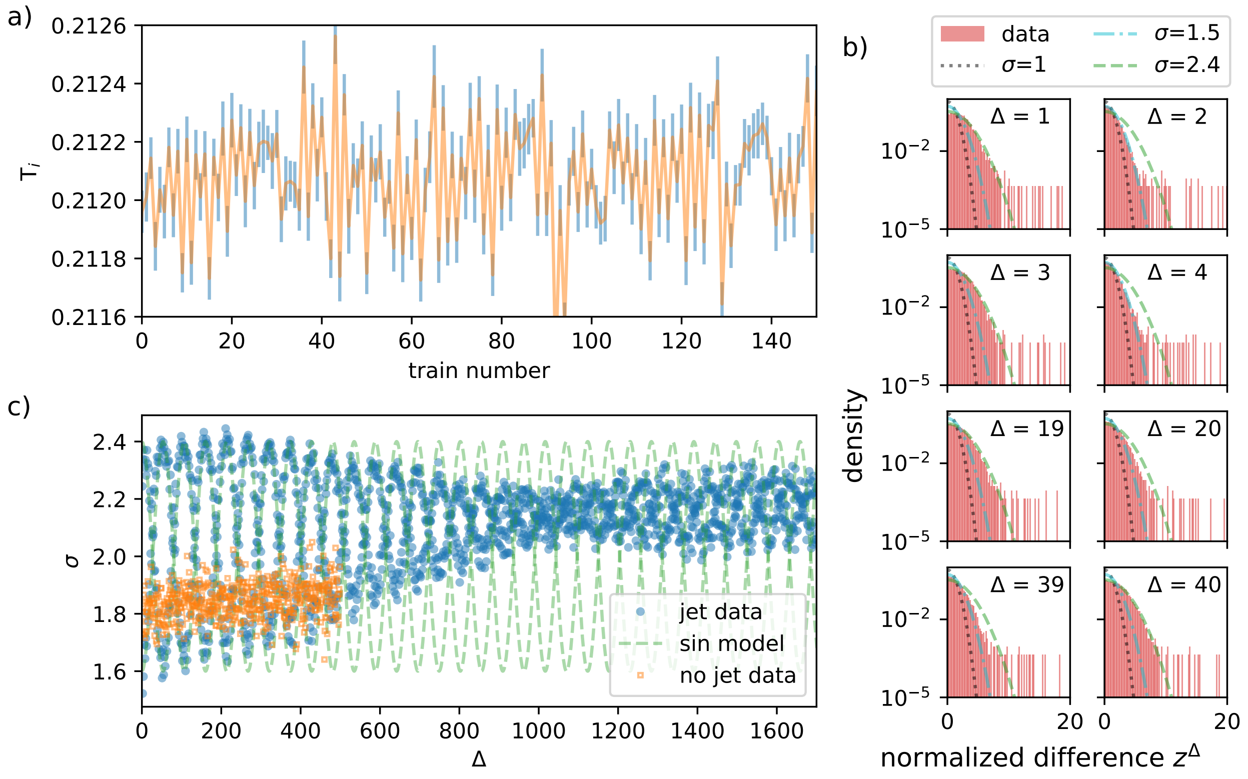

Figure S5: (a) Train-resolved transmission for 150 consecutive trains in orange with standard errors in blue. (b) Series of histograms of normalized transmission differences for a given train delay Δ. The expected distribution in the absence of excess noise (σ=1) is shown as the dotted black curve. Cases with excess noise of σ=1.5 and σ=2.4 are shown as dash-dotted cyan curves and green dashed curves, respectively. (c) Fitted excess noise as a function of the train delay Δ showing a frequency beating behavior together with a simple sin model (dashed green curve).

To characterize this effect in more detail, we compute the normalized differences z_i^Δ using:

$$z_i^\Delta = \frac{|T_i - T_{i+\Delta}|}{\sqrt{\sigma_i^2 + \sigma_{i+\Delta}^2}}, \tag{1}$$

where Δ is the delay between trains we consider, T_i is the I_0-weighted mean transmission of the train i and σ_i is the I_0-weighted standard error. If all T_i are measuring the same jet thickness, then z_i^Δ should be normally distributed with a standard deviation of unity. The resulting histograms for certain Δ values are shown in Fig. S5b. We also plot the ideal case of purely normally distributed z_i^Δ as a black dotted line labeled $\sigma = 1$. For all train delay

Δ, the normalized differences are noisier than expected. This itself is not very informative, as there could be many additional sources of noise. If we fit the distribution for $\Delta = 1$, we find an excess noise relative to the ideal case of $\sigma = 2.4$. Interestingly, if we do the same for $\Delta = 2$, we find the relative excess noise to be smaller at $\sigma = 1.5$. This alternating pattern repeats itself for $\Delta = 3$, 4, up to 19 and 20. Eventually, it switches phase as shown for $\Delta = 39$ and 40. This continues further, as shown in Fig. S5c where we plot the fitted excess noise σ for Δ up to 1700 showing that several beating patterns are visible. A shorter data set acquired without the liquid jet running in the beam shows no presence of oscillations, as shown in Fig. S5c by the orange square data points. The excess noise in this case is around $\sigma = 1.8$, but this could be due to detector saturation which was seen in 66% of the shots. In this condition, the intensity of the three beams on the detector was also unbalanced. To conclude, although we do observe thickness instabilities of the jet with this nozzle, the setup is overall quite stable and allows measuring dilute samples in transmission.

Data acquisition and real-time data analysis

The data acquisition and real-time data analysis were carried out in Karabo, the control and data acquisition system of the European XFEL.[9,10] During energy or delay scans, detector and control data were processed in real time to generate a preview of the transient XAS spectrum, supporting continuous monitoring and run-time decisions, such as verifying alignment and timing, and focusing the scan range around emerging spectral features. Relative to the capabilities previously reported in Ref. 5, the real-time data analysis was expanded to include new features. A detector-saturation history was introduced which records per-train saturation metrics, allowing the x-ray intensity on the detector to be adjusted live so that it is neither too low nor too high. Typically, we aim to have a few percent of the shots on the detector saturated. Additionally, the previously described normalization scheme, with two reference beams and one transmitted beam, was implemented in the analysis pipeline for liquid jet experiments. These updates were integrated quickly and reliably, demonstrating

the adaptability of the underlying analysis infrastructure.

GPU acceleration

As described in detail in Ref. 5, two correction steps need to be applied after subtraction of dark pedestal values: flat field correction compensating for the properties of the EBOZ diffractive optics and a non-linear correction step compensating for the DSSC detector response. This process is necessary once the EBOZ optic is aligned in the beam and requires only a few minutes of data acquisition. Computing the correction functions—in particular, fitting the non-linear correction function—is quite time-consuming and requires typically about 8 hours of computation on a high-performance computing (HPC) cluster node such as the ones found in the Maxwell cluster.[11]

Given the computational intensity of this processing step, we explored speeding it up by utilizing data center GPUs available on Maxwell. To this end, we used CuPy[12] which enabled us to reuse the initial high-level implementation based on NumPy[13] and Dask,[14] and only required replacing select NumPy calls with CuPy equivalents. With minor modifications to mitigate data transfer overhead and limit memory usage, we were able to reduce the runtime of the corrections processing drastically. As measured on a Maxwell node with a Tesla V100 GPU connected via PCIe and with 32 GB of GPU memory, the runtime of processing in one case was reduced from approximately eight hours to approximately 30 minutes. This speedup allowed us to move the processing from batch jobs executed on the Maxwell cluster to interactive sessions running on the online cluster hardware close to the beamline, further decreasing turnaround time.

Static Spectrum and Background Subtraction

In order to generate the solvent-subtracted spectra presented in Figure 2 of the main text, the water background must be removed. Figure S6a shows the total liquid jet absorbance from the sample as the blue curve, and the Co absorbance is a clear peak centered at 781.6

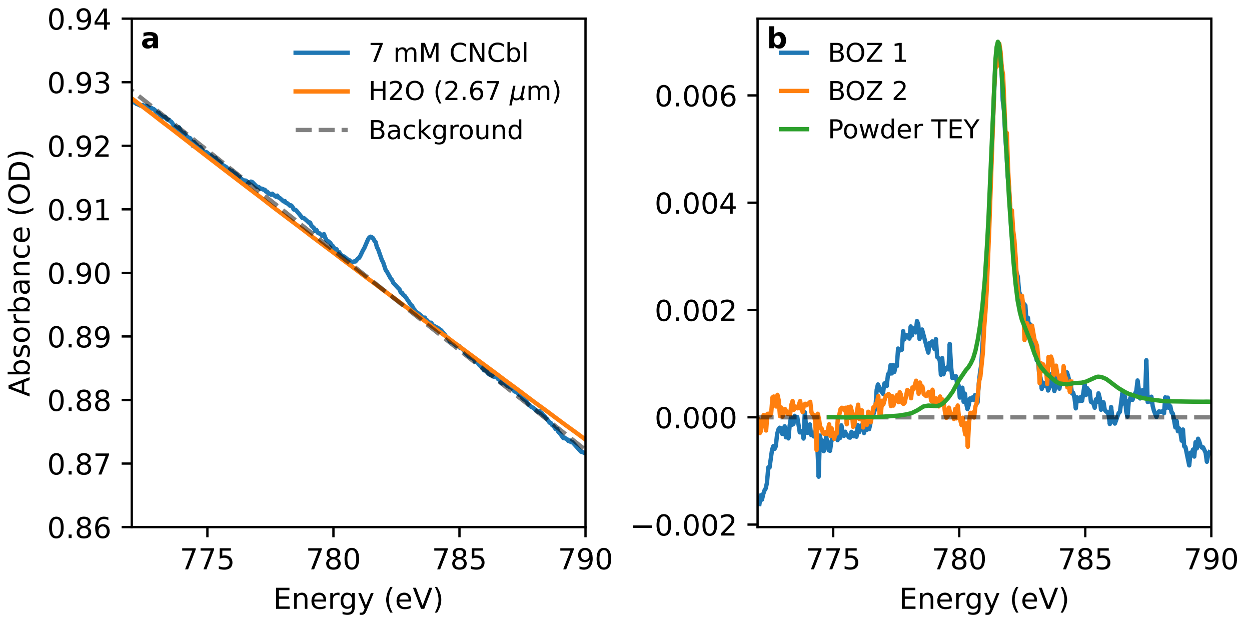

Figure S6: (a) Total sample and background absorbance. The water absorbance for a 2.67 μm thick water jet is plotted based on the tabulated cross section. The dashed background is used to generate the subtracted spectrum. (b) Background-subtracted spectra of CNCbl measured with two different zone plates are plotted together with the powder spectrum measured in TEY mode.

eV as shown in the main text. The orange curve gives the reference absorbance of water based on a 2.67 μm thick film generated using tabulated transmission data. There is a slight difference in the measured and modeled H_2O background, so a linear background (dashed black line) was used to generate the final steady state XAS instead of the reference water data.

It can also be seen that the measured spectrum exhibits some background structure as a function of incident energy. The background intensity modulation is most significant at ~778 eV and appears regardless of the measurement settings explored during the beamtime. This included the presence of the optical laser, the FEL repetition rate, and the X-ray pulse energy. It is noted that the background features are much greater in amplitude than the laser-induced change in absorbance, and the background is fully suppressed when computing the transient XAS signal. In subsequent experiments, we have successfully removed such features from the static spectra by measuring the solvent-only spectra and subtracting them

from the sample spectra.

Static absorption spectra of cyanocobalamin are shown in Figure S6b. The spectrum of 7 mM aqueous CNCbl is shown from measurements with multiple zone plates. BOZ 1 was the zone plate that was used for all transient spectra reported in this work. BOZ 2 was tested during alignment. BOZ 1 was used during the experiment because it gave the optimal I_t/I_0 intensity ratio for utilizing the full dynamic range of the detector. The background structure mentioned above produces peaks at 773.3, 778.2, and 787.3 eV. The intensities of background features are significantly greater for BOZ 1 confirming its instrumental origin. For comparison we have plotted the powder spectrum of CNCbl measured as the total electron yield (TEY) via drain current at the BOREAS beamline at the ALBA synchrotron. It can be seen that while both spectra exhibit a sharp L_3-edge feature with a high-energy shoulder at $\sim$782.75 eV, the low-intensity pre- and post-edge features visible in the powder spectrum may be obscured by the background in the solution measurement. However, there is no significant increase in pre-edge intensity for BOZ 2 where the background structure is less significant, so differences between the FEL and synchrotron spectra may also arise from fundamental differences in the detection mode.

Decay Modeling

The time delay traces shown in the main text were modeled using a sum of exponential functions that are convoluted with a Gaussian instrument response yielding an exponentially modified Gaussian. The data used in the fitting were the raw unscaled data shown in Figure S7. The signal-to-noise achieved during the experiment is such that the error bars on the ground state recovery are significantly smaller than on the excited state feature. The positive delay trace taken at 780 eV does not possess the S/N required for accurate analysis of the excited state dynamics. Fitting it alone can be achieved with either a single or two exponential functions, and this yields similar χ^2 values. Instead, the delay traces are modeled by a weighted global fit, which is dominated by the trace taken at 781.6 eV that

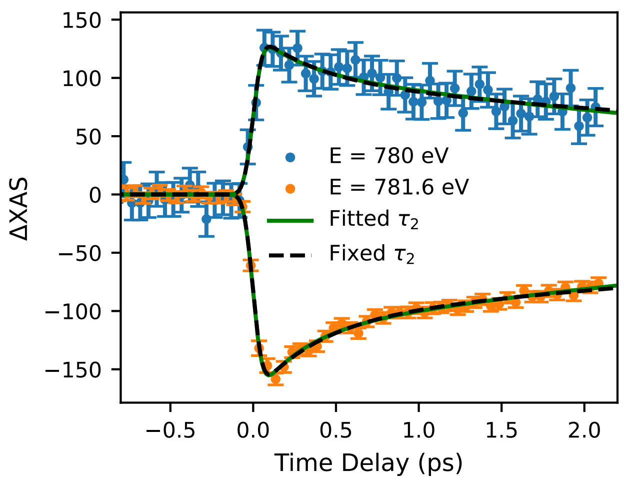

Figure S7: Unscaled delay scans taken at maxima of positive and negative spectral features. Weighted fits of the data are shown for the cases where the long time constant is either fixed (dashed black curve) to the 7.25 ps value determined by optical spectroscopy or allowed to float (green curve).

clearly shows two timescales. The ground state recovery is expected to be ∼7 ps based on previous X-ray and optical studies and is a function of both solvent and temperature. As the data is only collected to ∼2 ps, the error in fitting the longer timescale is very large. Here we performed fits with both a floating and fixed long time constant. The comparison assists in assessing the magnitude of the error in the value of the fast time constant. Both fits are shown in Figure S7 with parameters in Table S1. It can be seen that both fits are of very similar quality. The time constants change by 100 fs and 2.05 ps between the two fits. The 5.2 ps time constant is shorter than what is expected for ground state especially because the cold liquid jet is expected to slow this process. The 100 fs change in τ_1 is indicative of the magnitude of the uncertainty of the fitted value. Nevertheless, both fits are qualitatively consistent with previous ultrafast studies that identify a sub-500 fs cascade followed by ground state recovery, and the 800 ps spectrum can be associated with the S_1 state.

Table S1: Paramters used in fit of delay traces where τ_i (ps), A_i (μOD), and σ (ps) give the time constants, amplitudes, and width of the instrument response, respectively.

	τ_1	τ_2	A_{p1}	A_{p2}	A_{n1}	A_{n2}	σ
Clamped	0.39 (0.03)	7.25	42 (7)	97 (2)	-64 (3)	-108 (1)	0.044 (0)
Free	0.29 (0.05)	5.20 (0.68)	32 (9)	107 (5)	-56 (5)	-119 (5)	0.044 (0)

Excited State Spectrum Construction

In this section, we outline the procedure used to construct the spectrum of the excited state. The excited state spectrum (S_{ES}) at a given time delay may be constructed from the ground state spectrum (S_{GS}), the transient difference spectrum (ΔA), and the excitation fraction (f) according to

$$S_{ES} = \frac{\Delta A}{f} + S_{GS} \tag{2}$$

In the present work, the determination of the excited state spectrum is complicated by two details. First, the exact laser fluence on the sample is unknown due to significant drift in optical laser pointing from run to run. This precludes the possibility of calculating f from the absorption cross section. Instead, we use a transient difference spectrum measured at 200 fs to set reasonable bounds on the excitation fraction, then we construct the S_1 excited state spectrum from the 800 fs transient spectrum shown in the main text. Second, the background issues discussed above create a significant issue with the excited state spectrum because the transient difference indicates that the excited state features overlap the background feature around 778 eV. Here, we construct excited state spectra using two different ground state reference spectra. First, we use the liquid spectrum measured in the present experiment, but we set the baseline to 0 below 780 eV. Second, we construct the excited state spectrum from the powder spectrum shown in Figure S6b. Overall, it is demonstrated that the energies of the features we identify in the state spectrum are independent of both the ground state reference and the excitation fraction as long as they are chosen from the reasonable range identified below.

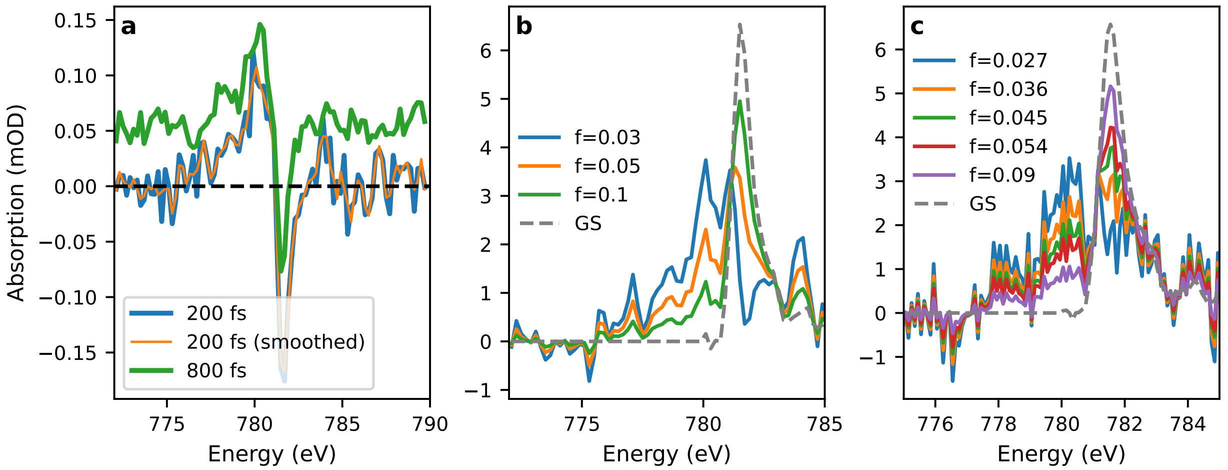

Figure S8: (a) Comparison of 800 fs spectrum (green top) with transient spectrum collected at 200 fs delay. The smoothed 200 fs spectrum used to generate the excited state spectrum is plotted in orange. (b) Excited state spectrum at 200 fs for excitation fractions between 2.5 and 10%. (c) Excited state spectrum at 800 fs for initial excitation fractions of 3, 4, 5, 6, and 10% following scaling to account for ground state recovery. The static spectrum following removal of the pre-edge is shown as a dashed gray line in parts (b) and (c).

Figure S8 shows the transient L-edge spectrum of CNCbl measured at 200 and 800 fs pump probe delay. The most significant difference observed between the 200 and 800 fs spectra is the relative amplitude of the positive and negative transient features. Overlaying the spectra and both times shows similar intensities for the positive features in the range 777 to 781 eV, but a greater amplitude in the loss feature at 781.6 eV for the 200 fs data. However, the 200 fs spectrum has a somewhat lower signal-to-noise, and the discussion of early time dynamics is left to future work. Here the 200 fs spectrum is used to set a limit on the possible excitation fraction of the sample. This is achieved by forming excited state spectra via Eq. 2 for excitation fractions between 3 and 10% as shown in Figure S8b. It can be seen that the 3% spectrum is completely unreasonable as indicated by the negative going feature at 781.6 eV (the energy of the loss feature in the transient spectrum). We conclude that the excitation fraction must be greater than this. At the other extreme, the 10% spectrum exhibits relatively small changes between the ground and excited state spectrum, and it is concluded that larger excitation fractions are unlikely and inconsistent

with theoretical predictions of the excited state spectrum.

Having identified a reasonable excitation fraction range at early times, the excited state spectrum at 800 fs is constructed for initial excitation fractions of 3, 4, 5, 6, and 10%. This percentage is further scaled by 0.9 to account for the fraction of molecules that have already returned to the ground state within 800 fs according to the 7.25 ps time constant taken for ground state recovery. This yields the final fractions shown in Figure S8c. It can be seen that the excitation fraction within this range only has a significant effect on the peak intensities and little effect on the energetic positions of features. A 5% spectrum is used for comparison between theory and experiment in the main text.

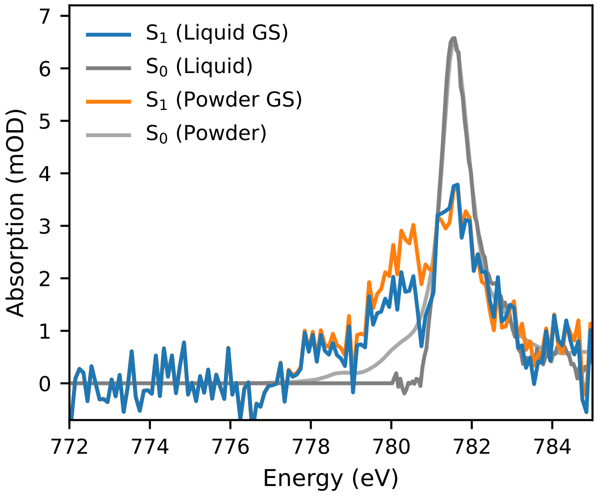

Figure S9: Comparison of the excited state spectra at 800 fs delay when constructed with either the static spectrum collected in the present experiment following background removal or the powder synchrotron spectrum. The excited state fraction at this delay is taken to be 4.5%.

Finally, we assess the effect of the background structure on the lineshape of the excited state spectrum. This is done by comparing the spectrum of the excited state at 800 fs generated from only data collected at SCS with the data of an excited state spectrum generated from the powder spectrum collected at the BOREAS beamline of the Alba synchrotron. Figure S9 shows the comparison between these two excited state spectra and includes the corresponding ground states. It can be seen that there is a significant intensity difference

in the 779 - 780.7 eV region of the spectrum. However, the key features of the spectrum remain. The excited state spectrum shows features centered at 778.2 and 780 eV regardless of the reference spectrum, and the most intense feature retains its energetic position and intensity regardless of the ground state spectrum used.

Theoretical Methods and Models

Structures

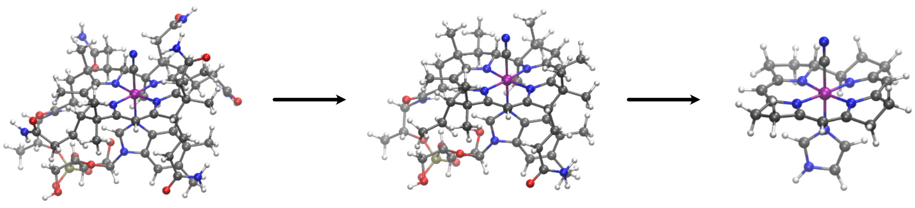

Figure S10: Cyanocobalamin was modeled starting with the geometry optimized crystal structure (left). This was reduced by removing side chains (center). CASSCF and TDDFT calculations were carried out on the $[ImCo(corrin)CN]^+$ model (right).

The structural models used for the ground and excited state of cyanocobalamin were obtained from density functional theory (DFT) calculations starting from the crystal structure. The sequence of structural models used is shown in Fig. S10. Firstly, the full crystal structure (CNCbl) is reduced by removal of amide side chains to give a model denoted by CNCbl-*red*. This is done to prevent non-covalent interactions between the amide groups and the CN^- ligand. It was found that these interactions lead to structurally distorted geometries, particularly in the excited state. Since the models do not include explicit solvent, such interactions are likely unphysical or overestimated. DFT optimizations are performed for the ground and excited state of CNCbl-*red*. Finally, the reduction to the $[ImCo(corrin)CN]^+$ model is performed by taking the core structure from the CNCbl-*red* and re-optimizing the positions of the hydrogen atoms. This model is similar to the model used extensively by Ko-

zlowski and coworkers, but it retains the corrin ring deformations and axial bond distances found in the more complete CNCbl-*red* model.

All calculations were performed with the 5.0.4 version of the Orca quantum chemistry package.[15] Geometry optimizations of the CNCbl-*red* structure were performed at the B3LYP/def2-TZVP level of theory. RIJCOSX was used with the de2/J basis set to accelerate calculations.[16,17] The continuum model (CPCM) for the solvent was used with the parameters for water. Dispersion forces were accounted for by the D3BJ method.[18] The structure of the S_1 state was optimized using the first TDDFT excited state. Coordinates of the CNCbl-*red* structures are provided below.

Table S2: Axial bond distances (Å) from DFT-optimized structures.

Structure	Method / Functional	Co–C (Å)	Co–$N_{Imidazole}$ (Å)
S_0	B3LYP	1.88	2.06
S_1	B3LYP	2.23	2.38
S_1[19]	BP86	2.21	2.27

CASSCF and AILFT

Table S3: Parameters from AILFT. All values are in eV.

	S_0	S_1
F^2_{dd}	10.1	10.0
F^4_{dd}	5.4	5.5
d_1	0.0	0.0
d_2	0.16	0.10
d_3	0.21	0.26
d_4	3.33	1.68
d_5	3.42	3.57
D_q	0.342	0.357
D_s	-0.016	0.244
D_t	0.031	0.183

CASSCF calculations were carried out on the $[ImCo(corrin)CN]^+$. The active space was chosen to include six electrons and the five 3d orbitals, CAS(6,5). The def2-TZVP basis set

was used together with the def2-TZVP/C fitting set, which was used for integral transformations. CASSCF calculations were carried out within the state-averaged formalism, and 5 quintets, 45 triplets, and 50 singlets included in the state-averaging. The *ab initio* ligand field theory (AILFT) procedure was used to extract ligand field parameters. Dynamic electron correlation was accounted for using the NEVPT2 treatment in the strongly contracted form. The results of the AILFT fitting to the CASSCF+NEVPT2 calculations are shown in Table S3. The 3d orbital energies (d_n) listed are the 1-electron eigen values of ligand field matrix. The values of the Dq, Ds, and Dt parameters result from a least square fit of these one electron energies to a D_{4h} symmetry.

Multiplet simulations

Table S4: Parameters used in multiplet simulations. All values are given in eV.

	S_0	S_1
F^2_{dd}	9.09	9.00
F^4_{dd}	4.86	4.95
F^2_{pd}	6.32	6.32
G^1_{pd}	5.95	5.95
G^3_{pd}	3.38	3.38
D_q	0.342	0.33
D_s	-0.016	0.244
D_t	0.031	0.183

The multiplet simulations were carried out using a crystal-field Hamiltonian that included along with crystal-field, electron-repulsion, and spin-orbit coupling evaluated for a model space including only the 2p and 3d orbitals. Two-electron interactions were parametrized in terms of the Slater-Condon parameters. The symmetry of the system was approximated as D_{4h} giving D_q, D_t, and D_s as ligand field parameters. The spin–orbit interaction was parameterized in term of ζ_{3d} and ζ_{2p}, which were set to the atomic values of 0.083 and 9.749 eV, respectively. All simulations were performed using the EDRIXS Python modules.[20] Simulated transitions are broadened with a Voigt profile that has a Lorentzian width of 0.43

eV to match the Co L_3-edge lifetime[21] and a 0.13 eV Gaussian width to match the 5000 resolving power predicted for the 2nd order operation of the SCS monochromator.[22] The parameters used in the multiplet simulations in Figure 3 of the main text are given in Table S4 For the core-hole states, the F^2_{pd}, G^1_{pd}, and G^3_{pd} were taken to be a 20% reduction from the atomic values. The choice of valence parameters was informed by the AILFT calculations. The Slater-Condon parameters that were used correspond to a 10% reduction from the AILFT values. The ligand field parameters were taken directly from the least squares fit of the one-electron eigen values of the AILFT ligand field matrix with the exception of the D_q parameter for the S_1 state, which was reduced by 0.027 eV relative to the AILFT value.

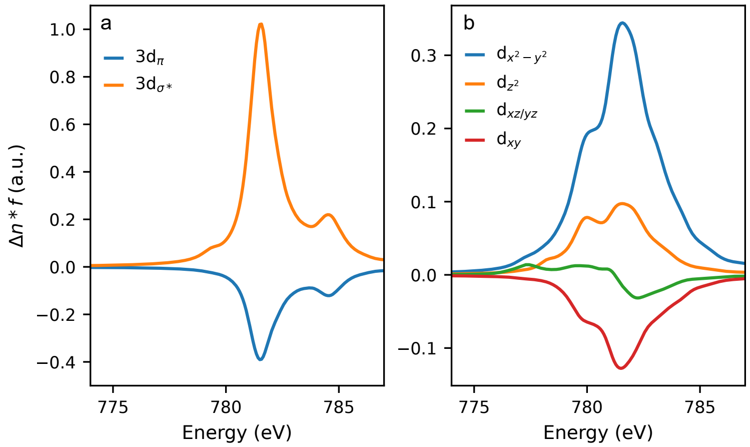

Figure S11: The orbital involvement is represented by the occupation number difference of the 3d orbital between the valence and core-excited state (Δn) multiplied by the transition intensity (f) for the (a) S_0 state and (b) S_1 state. For the nearly octahedral ground state, the individual d_π and $d_{\sigma*}$ contributions are summed.

The changes in the L_3-edge spectrum can be rationalized using the approach of Lundberg and coworkers, in which the changes in the 3d orbital energies and occupations are associated with the formation of the S_1 excited state.[23] This is demonstrated by the intensity-weighted orbital occupation difference spectra shown in Figure S11. These spectra are constructed transition-wise by multiplying the orbital population difference between the core-excited state ($2p^5 3d^7$) and the valence state ($2p^6 3d^6$) by the oscillator strength. Positive peaks indicate significant core to valence excitation character while negative peaks indicate that

the orbital is involved in double excitations. For example, the S_0 spectrum arises from $2p \rightarrow 3d(\sigma^*)$, but these transitions couple with d-d excitations ($d_\pi \rightarrow d_{\sigma^*}$). The negative $3d_\sigma$ curve in Figure S11a arises because the latter orbitals have lower population in the core-excited states due to double excitation character in the $2p^5 3d^7$ wavefunctions. This is similar for the S_1 case where the curve for the $d_{xz/yz}$ has nearly zero intensity up to 781 eV, but this does not mean that excitations from the 2p shell to this orbital do not contribute to the spectrum. Rather, this indicates that the $2p \rightarrow 3d_{xz/yz}$ excitations are balanced by the negative contributions of d-d excitations.

References

(1) Koralek, J. D.; Kim, J. B.; Brůža, P.; Curry, C. B.; Chen, Z.; Bechtel, H. A.; Cordones, A. A.; Sperling, P.; Toleikis, S.; Kern, J. F.; Moeller, S. P.; Glenzer, S. H.; DePonte, D. P. Generation and characterization of ultrathin free-flowing liquid sheets. *Nature Communications* **2018**, *9*, 1353.

(2) Ekimova, M.; Quevedo, W.; Faube, M.; Wernet, P.; Nibbering, E. T. A liquid flatjet system for solution phase soft-x-ray spectroscopy. *Struct. Dyn.* **2015**, *2*.

(3) SCS team BOZ calculator documentation. 2024; `https://rtd.xfel.eu/docs/bozcalc/en/latest/`.

(4) SCS team SCS Toolbox repository. 2024; `https://git.xfel.eu/SCS/ToolBox`.

(5) Le Guyader, L. et al. Photon-shot-noise-limited transient absorption soft X-ray spectroscopy at the European XFEL. *J. Synchrotron Rad.* **2023**, *30*, 284–300.

(6) Döring, F.; Rösner, B.; Langer, M.; Kubec, A.; Kleibert, A.; Raabe, J.; Vaz, C. A. F.; Lebugle, M.; David, C. Multifocus off-axis zone plates for x-ray free-electron laser experiments. *Optica* **2020**, *7*, 1007.

(7) Porro, M. et al. The MiniSDD-based 1-Megapixel Camera of the DSSC Project for the European XFEL. *IEEE Trans. Nucl. Sci.* **2021**, 1–1.

(8) Schlappa, J. et al. The Heisenberg-RIXS instrument at the European XFEL. *J. Synchrotron Rad.* **2025**, *32*, 29–45.

(9) Göries, D. et al. The Karabo SCADA System at the European XFEL. *Synchrotron Radiat. News* **2023**, *36*, 40–46.

(10) Carinan, C. et al. Real-time experiment steering at European XFEL within Karabo. Proc. ICALEPCS2025. 2025; pp 1701–1705.

(11) DESY Documentation for the Maxwell HPC Cluster. 2024; `https://docs.desy.de/maxwell/` [Accessed: 2024-07-24].

(12) Okuta, R.; Unno, Y.; Nishino, D.; Hido, S.; Loomis, C. CuPy: A NumPy-Compatible Library for NVIDIA GPU Calculations. Proceedings of Workshop on Machine Learning Systems (LearningSys) in The Thirty-first Annual Conference on Neural Information Processing Systems (NIPS). 2017.

(13) Harris, C. R. et al. Array programming with NumPy. *Nature* **2020**, *585*, 357–362.

(14) Dask Development Team Dask: Library for dynamic task scheduling. 2016.

(15) Neese, F. Software update: The ORCA program system—Version 5.0. *Wiley Interdiscip. Rev. Comput. Mol. Sci.* **2022**, *12*, e1606.

(16) Weigend, F.; Ahlrichs, R. Balanced basis sets of split valence, triple zeta valence and quadruple zeta valence quality for H to Rn: Design and assessment of accuracy. *Phys. Chem. Chem. Phys.* **2005**, *7*, 3297–3305.

(17) Weigend, F. Accurate Coulomb-fitting basis sets for H to Rn. *Phys. Chem. Chem. Phys.* **2006**, *8*, 1057–1065.

(18) Grimme, S.; Ehrlich, S.; Goerigk, L. Effect of the damping function in dispersion corrected density functional theory. *J. Comp. Chem.* **2011**, *32*, 1456–1465.

(19) Lodowski, P.; Jaworska, M.; Andruniów, T.; Garabato, B. D.; Kozlowski, P. M. Mechanism of the S1 excited state internal conversion in vitamin B12. *Phys. Chem. Chem. Phys.* **2014**, *16*, 18675–18679.

(20) Wang, Y.; Fabbris, G.; Dean, M. P.; Kotliar, G. EDRIXS: An open source toolkit for simulating spectra of resonant inelastic x-ray scattering. *Comput. Phys. Commun.* **2019**, *243*, 151–165.

(21) Krause, M. O.; Oliver, J. H. Natural widths of atomic K and L levels, Kα X-ray lines and several KLL Auger lines. *J. Phys. Chem. Ref. Data* **1979**, *8*, 329–338.

(22) Gerasimova, N. et al. The soft X-ray monochromator at the SASE3 beamline of the European XFEL: from design to operation. *J. Synchrotron Rad.* **2022**, *29*, 1299–1308.

(23) Pinjari, R. V.; Delcey, M. G.; Guo, M.; Odelius, M.; Lundberg, M. Restricted active space calculations of L-edge X-ray absorption spectra: From molecular orbitals to multiplet states. *J. Chem. Phys.* **2014**, *141*, 124116.

XYZ Coordinates

Reduced model of S_0

```
Co 4.30614573739636    2.48910343381351    5.07066987160068
C  4.02518102710398    2.96659917211235    3.27203295855601
N  3.86320448271912    3.24904172845182    2.16038622799577
N  2.52909208354184    2.97015892517164    5.51487997680525
N  3.71274811382816    0.69923720281168    4.63929942921908
N  6.18103842343575    2.18507649624963    4.69283179522916
N  4.65637611897405    4.31363369755091    5.45512831754017
C  2.33244356222859    4.29006300986013    6.17869111787030
C  0.84508672762619    4.63695761117787    5.78704266469322
```

C	0.24834791109436	3.22135012766564	5.62815627395171
H	-0.58319692820862	3.20342579085023	4.92795483102426
C	1.42176661074393	2.39094723402224	5.18950371197775
C	1.31488415564151	1.10523940768096	4.54967091982462
C	2.41113960887341	0.30144582750523	4.38631908183590
C	2.44227051684300	-1.13916380177905	3.84465531374891
C	3.84157508837973	-1.60671872982791	4.35078658278689
H	4.31427944522282	-2.28511646013041	3.63973240482238
C	4.57270028408286	-0.30155967109466	4.42364942198645
C	5.94016969689330	-0.17794105212382	4.25312995935928
H	6.47488953190132	-1.08120362648264	4.00499401625630
C	6.66758557815590	0.99845372873249	4.31343793999795
C	8.11921074678740	1.11141354549112	3.89013051390316
C	8.52181291910578	2.45075481679115	4.56740094132938
H	9.14579703366921	3.02838796483045	3.88614194509885
C	7.18007480244532	3.13259393193860	4.77174350484752
C	7.03231070200465	4.45740336714188	5.09670898355194
C	5.74340884415519	5.01639693176739	5.42510103071841
C	5.42614071599137	6.50147075417412	5.68247822364912
C	3.97260897505104	6.40584891941424	6.24739399247824
H	4.06133935023130	6.18630798147182	7.31127491215259
C	3.44719249170014	5.15180006997967	5.56231869843513
H	3.15581944721281	5.38883093780251	4.53830899642810
C	2.52004223563566	4.13839011664269	7.68914386312801
H	3.54323922700908	3.85593992127916	7.91745862344099
H	1.85676322296404	3.37127683083873	8.08659636562082
H	2.30405224698830	5.07754408158215	8.19505010846794
C	0.07033550441918	5.44378218272629	6.82695608533898
H	0.51291118693359	6.42672242345998	6.98763983636287
H	0.01754794480523	4.93038076282457	7.78586006163437
H	-0.95157693364285	5.59553156228431	6.47337667998393
C	0.74677416947544	5.34297245583081	4.42716515837687
H	1.15814319644266	6.35095589083236	4.46258586120467
H	1.26170059668838	4.78819983339913	3.64096530042197
C	-0.06898189814006	0.68826826147850	4.11276066559787
H	-0.03717500986141	-0.05334754120195	3.32385975442995
H	-0.61325056983028	1.54414795714118	3.71649494600664
H	-0.65912373163905	0.28029895243198	4.93828701414476
C	1.31822856159020	-2.07339199893803	4.30345435824031
H	1.09087838570967	-1.95638864546465	5.36210452032331
H	1.62475248525743	-3.10641577833685	4.13138998651457
H	0.40316934864740	-1.91492883600494	3.74056289321593
C	2.48156102790323	-1.07441928801432	2.30485967490036
H	3.32443372271110	-0.47424380668335	1.95761163733342
H	2.58804600532603	-2.08301034754405	1.90106864150901

C	3.79757226796085	-2.28353652699495	5.74107014266657
H	3.14475572148159	-1.70315917686939	6.39207065332891
H	3.34033246720669	-3.26496261798049	5.61022086190443
C	5.15726564045768	-2.43255971674045	6.41518051969249
H	5.62770177415804	-1.45857988270240	6.55323941371017
H	5.84171071920713	-3.01002479266963	5.78689147403642
C	5.18352652102910	-3.08444450973751	7.78575180260761
O	6.23547249145575	-3.12696484788575	8.42829753038065
N	4.04293924298514	-3.61910588303231	8.25505584097484
H	4.03016086811907	-3.99842172754337	9.18895359858839
H	3.16253825497088	-3.50718570547541	7.78255860249582
C	8.12858644333116	1.30092443437286	2.35712349616332
H	9.15506223740529	1.45229972949397	2.01854018442925
H	7.53622730634020	2.16978247737017	2.06476306340058
H	7.72242338035130	0.42042775464622	1.85777495119815
C	8.95689207947314	-0.11485113131470	4.24589582292202
H	8.95089424642134	-0.32580863671243	5.31450062378167
H	9.98718095317033	0.02808443767263	3.92254986873362
H	8.57484104799054	-0.99347980078141	3.72559690944980
C	9.25789378862271	2.33554130019481	5.92164680681802
H	9.03744542947551	3.22569721880563	6.51077736520292
H	8.84678794618614	1.50046292039917	6.49041645999753
C	10.77270413865644	2.21015925946680	5.78962563784080
H	11.23841802596402	2.15264887634931	6.77544217650841
H	11.07038183970250	1.32405990299051	5.23062026777934
C	8.25814849447610	5.34246643247836	5.11783977388496
H	9.08810507997324	4.87750989007344	4.59530648213708
H	8.59518510690445	5.56189563833589	6.13248935697678
H	8.07235123097827	6.29288916509027	4.62411438298801
C	5.45303704071277	7.23429486662058	4.32702434238049
H	6.43660905787728	7.17277940296874	3.86519991532785
H	5.21690657375937	8.28912300467822	4.46843017550790
H	4.72896685164065	6.81266152448661	3.62884740830806
C	6.34228331834960	7.23046411594829	6.67937503083021
H	5.86421408658887	8.18626835125259	6.91039477120329
H	7.29060460252418	7.47468884095511	6.20799022994022
C	6.61527915236048	6.48188134117653	7.98953072892612
H	5.70352101746276	6.37205565898102	8.57544541372463
H	6.99104093779014	5.47963521559930	7.78066650426343
C	7.67851868835390	7.20457538310479	8.79136642915733
O	8.85741372313759	7.22278293956929	8.42629756628694
N	7.24812667786658	7.85933177886581	9.88494418350370
H	6.29456942833739	7.72154332548077	10.17663122691302
C	3.12814424556090	7.65978775274068	6.08327581019255
H	2.89322360617189	7.85981489296820	5.03800387904503

H	2.18881154339165	7.56460922546450	6.62614247495950
C	8.13031397922911	8.58499480310332	10.77815402126129
H	8.89007285763147	9.09423834714429	10.18566391614061
H	7.53693172701629	9.33892307178835	11.29732021278940
C	8.81890464821530	7.69263368654442	11.80474488920555
H	9.39456701004842	6.92960662608501	11.27883171412632
C	9.72423931653777	8.48600506718206	12.72661325298840
H	9.15245382740583	9.24864086791434	13.26000391626882
H	10.51083860768919	8.97916499121114	12.15206040416831
H	10.19053513304642	7.82381768885525	13.45545545204682
P	7.84334347234693	5.46558235496541	12.87785552800738
O	7.72273254668402	4.94357326769698	11.31860407818641
O	7.77957025609202	7.04678006931110	12.57518642893348
O	9.17829885667754	5.04991490334650	13.40527117823850
O	6.59855407531890	5.11299097155012	13.62110559916436
C	6.92706082064821	1.74288308688636	9.83490544709114
H	7.05527518974297	0.80149563861651	10.37118120919743
C	6.53765567891928	2.87624422070336	10.80777780703831
H	6.11910036829502	2.46791282573906	11.72935229602999
C	7.88795039832984	3.57138872013417	11.02829185294052
H	8.45218685001068	3.06516633374275	11.81146486245974
C	8.58703495169969	3.39367408666657	9.68314649128140
H	8.24158182782223	4.16716695335073	8.99135593847297
C	10.09300381663752	3.45372702591537	9.72328405330358
H	10.48623856168858	3.25019694376259	8.72317306697473
H	10.37339855692101	4.47628625700077	9.99795001186575
O	8.14750801083489	2.10312932352702	9.22290728282240
O	5.61994597134668	3.76100381848812	10.19476898474778
H	5.85252987152398	4.65169477354625	10.50172396939295
O	10.59458043893002	2.51086751800389	10.66706745871814
H	11.55034241461852	2.62417500062909	10.72661376894155
N	5.91946430659391	1.51490571206608	8.81269005443602
C	5.81676664415414	2.16740263837677	7.63308751604383
H	6.57851141628444	2.83941573422991	7.28474214043787
N	4.69888282521614	1.87210971933110	6.99588641923007
C	2.73737636247740	0.40586845511651	7.72198288925354
H	2.10805554508375	0.57579905414915	6.86587612148654
C	2.29079796268830	-0.43053559539284	8.73484401578336
C	3.09141575332196	-0.67237975379724	9.88221197257069
C	4.33182204297553	-0.06155939802480	10.00827071605847
H	4.93802592398421	-0.22795357202163	10.88891368281796
C	4.76340745772977	0.76600731544827	8.97759916326258
C	3.99213208426127	0.99990282270654	7.82842320261373
C	0.95319522047986	-1.10373573419004	8.59108912112883
H	0.45900573910802	-0.78587611612987	7.67366967055222

H	0.29475930486637	-0.87825521665079	9.43356687101636
H	1.06299691410531	-2.19167361412612	8.55790767479658
C	2.59899180932344	-1.59083970745717	10.96520638969922
H	3.32949764207219	-1.67172771761838	11.76948830514532
H	2.40851262150270	-2.59357439471044	10.57403607459803
H	1.65734714859464	-1.23539665547172	11.39183365522494
H	1.56519305068618	-0.63919933463085	1.90636821373567
H	-0.30471828763888	5.42158575631429	4.14587637039532
H	3.64957871019809	8.52972638884028	6.48628532957836
H	-0.12290825112883	2.82513714760132	6.57910372538037
H	11.18144977258554	3.08319480188498	5.27489384600808

Reduced model of S_1

Co	4.34238162636515	2.48612850784750	5.20755454026126
C	3.93729361576495	2.99868727082506	3.07281114581297
N	3.76681724423902	3.25793236574907	1.95174000281923
N	2.57039331908588	2.92032540836452	5.70614871861964
N	3.75517780308310	0.65516686788160	4.80039562329036
N	6.20879846637745	2.18466234473734	4.89458240393589
N	4.68480809403800	4.27101843190407	5.60877423775503
C	2.37482438726933	4.22496574690663	6.39014583569365
C	0.87881830623242	4.56441055144950	6.03410668185164
C	0.29011187139816	3.14156494891129	5.89343889104011
H	-0.55320442483838	3.11381517373124	5.20730797611218
C	1.46203041548178	2.31898084261651	5.43762151768503
C	1.34922278958423	1.00838552454521	4.83077470990117
C	2.44160527592757	0.21554545147731	4.63640726729845
C	2.48344188138214	-1.24313913410504	4.14972728969336
C	3.91336671048495	-1.65888460705209	4.61960015280125
H	4.38655005383838	-2.34272399224056	3.91349058819048
C	4.60770498380716	-0.33489725907173	4.63791411142216
C	6.00088702329741	-0.19590135210283	4.46338539328548
H	6.53467233366957	-1.10116196688189	4.21878107312281
C	6.71645268089765	0.95929572384930	4.52756623391008
C	8.17710617349693	1.08364898224842	4.15567672364871
C	8.55831699696581	2.39641692582933	4.89404379167415
H	9.21480311769699	2.99284886318913	4.26183247895087
C	7.21217448828811	3.07417246285593	5.05086277117286
C	7.06453896177052	4.42432840360292	5.38608503419927
C	5.78577638048451	4.97959353081564	5.66907544343319

C	5.46802126886449	6.45077183312343	5.97939083208638
C	4.00812754522892	6.33651868449616	6.51588526509299
H	4.08251495634090	6.08137302797634	7.57267073503429
C	3.48294833518045	5.10886768724000	5.78153753801843
H	3.17447826948584	5.38535894381775	4.77108993422471
C	2.58726400383458	4.03657027938624	7.89425941011514
H	3.61065422934777	3.73373172760230	8.09541001375015
H	1.92286151861637	3.26510534346026	8.28118130715641
H	2.38599814491237	4.96312581714295	8.42837189960624
C	0.11903874435638	5.36610919596555	7.08879377030394
H	0.54859010201569	6.35767896262601	7.22933277647014
H	0.09994191451801	4.85959552745294	8.05264297727074
H	-0.91433305205577	5.49780770655500	6.76150205172524
C	0.75188465979560	5.26787369146258	4.67649223861909
H	1.14841808192305	6.28241571633443	4.71022292549882
H	1.26783041777595	4.72247155720215	3.88425502112324
C	-0.04827522829715	0.55794721240485	4.48419021427638
H	-0.04794307673260	-0.21273815773698	3.72317739583951
H	-0.62493203165566	1.39247790188473	4.08882820185939
H	-0.58449315252645	0.17536479906042	5.35723173580377
C	1.39882641985615	-2.18126708488146	4.68795282011140
H	1.20716655571101	-2.02640624381984	5.74867685513744
H	1.72084137976837	-3.21389506770282	4.54482735346759
H	0.46114808891996	-2.06303078890495	4.15292679518374
C	2.46151945699224	-1.24384446877530	2.60792096873399
H	3.27442232371383	-0.63968323593204	2.20142518868502
H	2.57549543076336	-2.26599154070454	2.24245382894029
C	3.93253322405584	-2.29575096634863	6.03129323095126
H	3.27321996428148	-1.71666427006207	6.67658697422433
H	3.50695608436395	-3.29520972334696	5.93943041052278
C	5.30911332299497	-2.37739280248440	6.68236785454852
H	5.75082046329718	-1.38510624887804	6.78322792747847
H	6.00137486748325	-2.95407448407455	6.06251147297716
C	5.37825770627681	-2.98057835140748	8.07447469319487
O	6.44671125685723	-2.98534273085634	8.69017421952822
N	4.25785312159478	-3.51399343014589	8.59110209425111
H	4.27169966397579	-3.84372880843064	9.54366787575389
H	3.36200512243585	-3.41948107057053	8.14439034986391
C	8.23980004428888	1.33679788506729	2.63135363521458
H	9.27756309085268	1.50298425251012	2.33825635726200
H	7.65544008004512	2.21473535105055	2.35138632355209
H	7.85423847161569	0.47477599746890	2.08614318218927
C	9.00438861279810	-0.15540904835188	4.48766853635571
H	8.96566402407244	-0.40811198782079	5.54637844858977
H	10.04309462005897	0.00229974904499	4.20083476209893

H	8.63965220098767	-1.01227134245189	3.92079496651032
C	9.23033393999752	2.23608220309588	6.27783247123239
H	8.98532942194137	3.10631092591843	6.88811872730844
H	8.79290897667387	1.38137728854133	6.79602946517444
C	10.74915345217029	2.11153192750443	6.20772706569848
H	11.16871974377544	1.99853271208561	7.20888581032615
H	11.06761342624814	1.25512090216251	5.61527911109034
C	8.29836436731909	5.28495045678471	5.45639734325750
H	9.13682369032089	4.81992661923341	4.94912313301846
H	8.60447492181338	5.47627449662362	6.48712725844862
H	8.13506736537321	6.25028859114849	4.98462742353941
C	5.51969575290364	7.23664699889210	4.65402080362518
H	6.51337734379174	7.19565167195845	4.21134981462911
H	5.27715596338512	8.28400903771360	4.83361683149037
H	4.81102900893217	6.84014847607611	3.92615950037360
C	6.36937381802911	7.13997778584890	7.01688173044926
H	5.89162465975179	8.08926438599076	7.27249335645545
H	7.32453904943733	7.39813972200871	6.56722255094511
C	6.62349415307706	6.35083415618968	8.30937254735997
H	5.71401268691115	6.26961919499518	8.90388786553310
H	6.95545936892546	5.33750202989170	8.08122534917344
C	7.72584427483835	7.02119623362049	9.10360936238050
O	8.90569932918466	6.95412708807659	8.74644906125830
N	7.33203963288302	7.73612999756074	10.17212755344249
H	6.36918387719801	7.67427404117486	10.45868792112560
C	3.16612635157990	7.59569464227891	6.38424975170686
H	2.93512272515485	7.82431357280120	5.34382063951772
H	2.22535083124902	7.48527229658358	6.92138797534259
C	8.25369193440582	8.44835009872880	11.03617285274115
H	9.01934601468613	8.92196177793911	10.42162633936695
H	7.69376264252384	9.23022091213193	11.55081354262546
C	8.93311403168681	7.55449196346895	12.06723809498523
H	9.47381046661912	6.76358382496344	11.54588865831528
C	9.88045008248196	8.33751478038361	12.95509402973634
H	9.34161051266401	9.12419165855990	13.48783658547976
H	10.66655292973247	8.80002841639071	12.35488093472786
H	10.34566492719396	7.67543325262854	13.68463456787491
P	7.91920578951977	5.38094214791190	13.20515147882047
O	7.74115786575571	4.82283597621566	11.66433966795288
O	7.88964599641336	6.95630385507483	12.86946380277558
O	9.25665568231420	4.94117598983175	13.70537653818187
O	6.68687400220058	5.08103344316124	13.99014284140105
C	6.96556386554585	1.57005977502073	10.27073391151675
H	7.09061043666367	0.64914422308556	10.84236741033457
C	6.56383517193611	2.73760868832509	11.20019351371781

H	6.13894890852090	2.36086904188837	12.13244803561763
C	7.90936736433668	3.44478002231536	11.40292491909361
C	6.56383517193611	2.73760868832509	11.20019351371781
H	6.13894890852090	2.36086904188837	12.13244803561763
C	7.90936736433668	3.44478002231536	11.40292491909361
H	8.47992176748383	2.95765448832423	12.19349722388379
C	8.60082000498783	3.23613198385152	10.05966870211925
H	8.22004810040032	3.96829052873857	9.34085312014938
C	10.10315190546665	3.35065170856288	10.08260257192910
H	10.49727247036694	3.14169716742432	9.08411323060958
H	10.34599122069595	4.38826287820754	10.33477521292398
O	8.19802872708703	1.91322518723923	9.66493370826194
O	5.64817266176582	3.59864648704098	10.54901913410758
H	5.86754632642422	4.49836633606699	10.83828263620179
O	10.64631212450269	2.44713524009339	11.04114537827316
H	11.58867010592494	2.63166037841797	11.12856769577281
N	5.97761613499524	1.31009870717128	9.24055455262678
C	5.89287072626666	1.94232068091451	8.03774288991153
H	6.67641369305027	2.58665246269994	7.68284258897082
N	4.77740125658757	1.67067119478956	7.40034126085427
C	2.78360596128853	0.26899542029121	8.11568216400979
H	2.18424896409735	0.44527482830671	7.23637908306715
C	2.29621166834779	-0.54307218293192	9.13006725853191
C	3.06959242194591	-0.78421769055251	10.29677667178840
C	4.32305945749356	-0.20222067125284	10.44256215046918
H	4.90644675945901	-0.37721645215984	11.33729638886978
C	4.79813154916654	0.59815294899547	9.40957304796025
C	4.04940040323073	0.83495266427726	8.24343672228914
C	0.95018421292842	-1.19530655659175	8.96774803046198
H	0.48373820063561	-0.88832363660525	8.03209416841365
H	0.27495118386008	-0.94095491228950	9.78860405208145
H	1.04036480601135	-2.28562908838664	8.95920691575172
C	2.53949428353173	-1.68570129768981	11.37706217054957
H	3.24451944172894	-1.75722281958324	12.20467376752772
H	2.36033497674796	-2.69425859221641	10.99376161335095
H	1.58540892100284	-1.32406342102913	11.76918165855529
H	1.51873512554231	-0.84647958811545	2.23216660954457
H	-0.30345071321667	5.33119943798919	4.40606635967702
H	3.68607963418951	8.45327099682510	6.81451287134366
H	-0.06169467231191	2.74782056575587	6.85279025826864
H	11.18295720116687	3.00882034310292	5.76014981293882
